# Targeting integrated stress response with ISRIB combined with imatinib treatment attenuates RAS/RAF/MAPK and STAT5 signaling and eradicates chronic myeloid leukemia cells

**DOI:** 10.1186/s12885-022-10289-w

**Published:** 2022-12-02

**Authors:** Wioleta Dudka, Grazyna Hoser, Shamba S. Mondal, Laura Turos-Korgul, Julian Swatler, Monika Kusio-Kobialka, Magdalena Wołczyk, Agata Klejman, Marta Brewinska-Olchowik, Agata Kominek, Milena Wiech, Marcin M. Machnicki, Ilona Seferynska, Tomasz Stoklosa, Katarzyna Piwocka

**Affiliations:** 1 Laboratory of Cytometry, Nencki Institute of Experimental Biology, Polish Academy of Sciences, 3 Pasteur Street, 02-093 Warsaw, Poland; 2 Center of Postgraduate Medical Education, Laboratory of Flow Cytometry, Warsaw, Poland; 3 Laboratory of Bioinformatics, Nencki Institute of Experimental Biology, Polish Academy of Sciences, Warsaw, Poland; 4 Laboratory of Animal Models, Nencki Institute of Experimental Biology, Polish Academy of Sciences, Warsaw, Poland; 5 Department of Tumor Biology and Genetics, Medical University of Warsaw, Warsaw, Poland; 6 Department of Hematology, Institute of Hematology and Blood Transfusion, Warsaw, Poland

**Keywords:** ISR, ISRIB, CML, Myeloid leukemia, TKI resistance, STAT5, RAS/RAF/MAPK, PTPN11

## Abstract

The integrated stress response (ISR) facilitates cellular adaptation to unfavorable conditions by reprogramming the cellular response. ISR activation was reported in neurological disorders and solid tumors; however, the function of ISR and its role as a possible therapeutic target in hematological malignancies still remain largely unexplored. Previously, we showed that the ISR is activated in chronic myeloid leukemia (CML) cells and correlates with blastic transformation and tyrosine kinase inhibitor (TKI) resistance. Moreover, the ISR was additionally activated in response to imatinib as a type of protective internal signaling. Here, we show that ISR inhibition combined with imatinib treatment sensitized and more effectively eradicated leukemic cells both in vitro and in vivo compared to treatment with single agents. The combined treatment specifically inhibited the STAT5 and RAS/RAF/MEK/ERK pathways, which are recognized as drivers of resistance. Mechanistically, this drug combination attenuated both interacting signaling networks, leading to BCR-ABL1- and ISR-dependent STAT5 activation. Consequently, leukemia engraftment in patient-derived xenograft mice bearing CD34+ TKI-resistant CML blasts carrying *PTPN11* mutation responsible for hyperactivation of the RAS/RAF/MAPK and JAK/STAT5 pathways was decreased upon double treatment. This correlated with the downregulation of genes related to the RAS/RAF/MAPK, JAK/STAT5 and stress response pathways and was associated with lower expression of STAT5-target genes regulating proliferation, viability and the stress response. Collectively, these findings highlight the effect of imatinib plus ISRIB in the eradication of leukemic cells resistant to TKIs and suggest potential clinical benefits for leukemia patients with TKI resistance related to RAS/RAF/MAPK or STAT5 signaling. We propose that personalized treatment based on the genetic selection of patients carrying mutations that cause overactivation of the targeted pathways and therefore make their sensitivity to such treatment probable should be considered as a possible future direction in leukemia treatment.

**Supplementary Information:**

The online version contains supplementary material available at 10.1186/s12885-022-10289-w.

## Background

Chronic myeloid leukemia (CML) driven by oncogenic BCR-ABL1 tyrosine kinase can be successfully treated with molecular targeted therapy [1, 2]. However, although imatinib shows remarkable clinical efficacy in the chronic phase (CML-CP), the effects in advanced phases (CML-BC) are short-lived, complete remissions are rare, and relapse often occurs [3–5]. Many patients show primary or secondary resistance to imatinib or second-generation tyrosine kinase inhibitors (TKIs), such as dasatinib, nilotinib and bosutinib. The resistance originates in the majority of cellular intrinsic mechanisms, either mediated directly by BCR-ABL1 point mutations, which are predominant in primary resistance, or by activation of BCR-ABL1-independent signaling pathways, often responsible for recurrence of the disease and therapy relapse [6, 7]. The most recognized pathways responsible for resistance include JAK2/STAT5, RAS/RAF/MAPK and PI3K/Akt/mTOR [5, 8, 9]. They increase proliferation, the anti-apoptotic response and survival, and cytokine and growth factor signaling, which collectively promote resistance to treatment and disease progression.

Among others, these resistance-promoting pathways can be specifically activated by pathogenic *PTPN11* gain-of-function gene mutations, which have been identified in myeloid malignancies, including tyrosine kinase inhibitor (TKI)-resistant CML and AML [10]. The *PTPN11* gene encodes the nonreceptor protein tyrosine phosphatase SHP2, which is required for the activation of the RAS/RAF/ERK, JAK/STAT and PI3K/AKT pathways in response to growth factors and cytokines. *PTPN11* mutations, which block autoregulation of SHP2 catalytic activity and lead to hyperactivation of RAS/MAPK, JAK/STAT, PI3K/AKT signaling, are responsible for the development of the prosurvival and resistant phenotype in myeloid leukemias and are associated with an overall poor prognosis*.* Therefore, targeting the prosurvival signaling pathways that promote the resistance phenotype is one of the current strategies for eradication of resistant cells [11–13].

Previously, we showed that the PERK-eIF2α pathway, which is part of the integrated stress response (ISR), is activated in CD34+ CML-BP cells and is correlated with disease progression and resistance to TKIs [14]. The ISR is a highly conserved signaling pathway responsible for cell adaptation and survival under stress conditions [15–18]. This is achieved by phosphorylation of the eukaryotic translation initiation factor eIF2α that attenuates the guanine nucleotide exchange factor eIF2B, leading therefore to remodeling of translation and transcription of stress response effector genes, including CHOP and GADD34, which are ISR markers [19]. The ISR is one of the mechanisms sustaining homeostatic balance in healthy cells under physiological conditions; however, cancer cells can utilize the ISR to survive and develop drug resistance. Moreover, we and others have shown that the ISR can be additionally activated by chemotherapy as a protective signaling pathway [14]. Although previous reports demonstrated that the ISR is active in metastatic solid tumors [20], it has not been deeply investigated in blood disorders. We have observed that the ISR is active in myeloid leukemia blast cells and additionally activated in response to imatinib as an adaptive protective mechanism [14]. In addition to our studies, although data is still limited, the regulatory connections between MAPK/STAT5, MYC, MEK (mitogen-activated protein kinase/ERK kinase) and ISR signaling have been implicated in leukemia [21–23]. Since it was recognized, the ISR has been proposed as a therapeutic target in cancer [24–26]. Nevertheless, no efficient, specific strategy has been proposed, especially for hematological malignancies.

We report here that inhibition of ISR signaling by the small molecule ISRIB combined with imatinib increases sensitivity to imatinib, eradicates CML-BP cells resistant to TKIs and decreases leukemia engraftment. We show that such double treatment specifically changes the gene expression profile and inhibits oncogenic RAS/RAF/MAPK/ERK and JAK/STAT5 signaling. Therefore, we propose the combination of ISRIB and imatinib as a possible therapeutic strategy to eradicate TKI-resistant leukemic cells exhibiting hyperactivation of the RAS/RAF/MAPK and STAT5 pathways, which can be successfully identified by NGS analysis of driver mutations, including *PTPN11*.

## Methods

### Cell culture

The K562 cells (CCL-243) and LAMA84 cells (CRL-3347) were purchased from American Type Culture Collection (ATCC) and cultured as previously described [14]. Cells were authenticated at ATCC service and were regularly tested for Mycoplasma contamination. Detailed description of the two-step generation of cells expressing the nonphosphorylable form of eIF2α is provided in the Supplementary Information.

### Isolation of CD34+ CML-BP patient cells

Patients’ material was obtained from the Institute of Hematology and Blood Transfusion in Warsaw, Poland, following informed written consent, in accordance with the Declaration of Helsinki and the guidelines for good clinical practice. All protocols were approved by the local Ethical Committee (Ethical and Bioethical Committee UKSW, Approval No. KEiB-19/2017, Approval No.WAW2/059/2019 and WAW2/51/2016). The characteristics of patient is detailed in the Supplementary Information. Peripheral blood mononuclear cells (PBMC) were isolated by density gradient centrifugation and CD34+ cells were separated using EasySep human CD34+ selection cocktail (StemCell Technologies, Inc.). CD34+ cells were short-term cultured in IMDM medium (Invitrogen) with 10% FBS, 1 ng/ml of granulocyte-macrophage colony-stimulating factor (GM-CSF), 1 ng/ml of stem cell factor (SCF), 2 ng/ml of interleukin-3 (IL-3). Cells were cryopreserved and kept in -180°C until usage.

### Cell treatment

Thapsigargin (Sigma) was used at 100 nM; imatinib (gift from Lukasiewicz Pharmaceutical Institute, Warsaw) was used at 0,5 or 1 μM concentrations in vitro or at given doses in vivo. ISRIB (Merck, SML0843) was used at 250 nM concentration in in vitro signaling studies or at given doses in vivo. GSK2656157 (GSK157) (Calbiochem) for in vitro test was dissolved in DMSO and given as indicated. For analysis of the effect of ISR inhibition on *GADD34* and *CHOP* mRNA levels, cells were pretreated for 2 hours with ISRIB or GSK157 inhibitors at given concentrations, followed by 2 hour treatment with 100 nM TG to activate ISR response. For analysis of signaling pathways, cells, if indicated, were treated for 2 hours with 250 nM ISRIB and/or, if indicated, 2 hours with thapsigargin to activate ISR. Then, 1 μM imatinib was added (with or without previous ISR induction by thapsigargin), and cells were analysed after 16 hour incubation. For in vivo studies, in the first step the general stock of GSK157 was made (53,3 g of GSK157 to 1523 μl DMSO). In the second step 20 μl of the general stock of GSK157 was added to 44 μl of PEG400 (MERC, #8074851000) and 40 μl of saline (not PBS).

### In vivo experiments

All in vivo experiments on mice (immunodeficient NOD.Cg-PrkdcscidIl2rgtm1WjL/SzJ mice), were performed in accordance with the Poland’s National Ethics Committee for Animal Experimentation and the Animal Protection Act in Poland (Directive 2010/63/EU). All protocols and experiments were carried out in relevant guidelines and regulations, and were approved by the Second Local Ethics Committee (Permission No. WAW/51/2016). Cells (10^6^) were injected subcutaneously or into tail vain. Mice were treated with: imatinib - twice a day (50 mg/kg); GSK157 - once a day (20 mg/kg); ISRIB - once a day (2 mg/kg) or in combination with the same doses, as indicated. Experimental schemes are presented as part of Figures. For calculation of drugs synergy in bone marrow engraftment, the CDI (coefficient of drug interaction) formula was used: CDI = AB/(A × B), where AB is the ratio of the 2-drug combination group to the control group and A or B is the ratio of the single drug group to the control group; CDI < 1 indicates synergism (CDI < 0.7 indicates a significantly synergistic effect); CDI =1 indicates additivity; CDI > 1 indicates antagonism.

### Flow cytometry

Apoptotic cell death was detected using Annexin V-PE Apoptosis Detection Kit I (BD Biosciences #559763) as described [14]. To detect phosphorylation of STAT5 and S6K, cells were incubated with eBioscience™ Fixable Viability Dye eFluor™ 455UV (Thermo Fisher) to discriminate dead cells, followed by staining using Transcription Factor Phospho Buffer Set (BD Pharmingen) and antibodies: anti-phospho-STAT5 (Tyr694)-PE, and anti- phospho-S6 (Ser235, Ser236) – eFluor450 (eBioscience, Thermo Fisher). Events were acquired using BD LSR Fortessa cytometer (Becton Dickinson) and then analysed by FlowJo Software (Becton Dickinson).

### Western blot

Western blot analysis was performed in a standard conditions, as previously described [14]. The full-length membranes were properly cut based on the protein marker size according to target protein sites into several parts prior to hybridization with primary antibodies, and every blot was then incubated with its primary antibody. List of antibodies is presented in the Supplementary Information (Table 1). Images of original non-cropped parts of membranes (after initial cut based on marker size prior the hybridization), together with different expositions, are presented in the Supplementary Information (Fig. S9).

### RT-qPCR analysis

Total RNA was extracted using TRI Reagent (Sigma #T9424) or by Renozol (Genoplast #BMGPB1100–2) followed by Total RNA Mini column purification kit (A&A Biotechnology #031–100). 2 μg of RNA was subjected to reverse transcription using M-MLV enzyme (Promega #M1705), dNTP mix 100 mM each (BLIRT #RP65) and oligo (dT)_18_ primers (Bioline #BIO-38029). The RT-qPCR reaction was performed using SensiFAST SYBR Hi-ROX Kit (Bioline #BIO-92020) on the StepOnePlus™ platform (Thermo Fisher Scientific) according to MIQE guideline. Primers sequences are listed in the Supplementary Information. The comparative 2^-ΔΔCt^ method was used to determine the relative mRNA level using StepOnePlus software. 18SrRNA was used as a reference control. Data are presented as mean values ± SD; *n* = 3–5. Statistical significance was assessed using unpaired Student’s t-test with Welch’s correction and *p* ≤ 0,05 was estimated as significant (*p ≤ 0.05; **p ≤ 0.005; ***p ≤ 0.001; ****p ≤ 0.0005).

### RNA sequencing and data analysis

RNA was isolated as described in RT-qPCR section. The library was prepared using NEB Next Ultra II Directional RNA library Prep kit for Illumina (#E7335S/L). Sample analysis: the quality of raw data was verified in FASTQ format from RNA-Seq experiments with FastQC [27]. Due to observed high quality of the raw data, no further processing of reads was performed. Data analysis was done using the SquIRE [28] pipeline. Human genome hg38 and corresponding refseq gene annotations were downloaded from UCSC (https://genome.ucsc.edu) [29] with SQuIRE. STAR version 2.5.3a [30], StringTie version 1.3.3b [31], and DESeq2 version 1.16.1 [32] were used within the SQuIRE pipeline for alignment of reads, transcript assembly and quantification, and differential gene expression analysis, respectively. Differentially expressed genes with false discovery rate (FDR) < 0.05 were reported here. Principal component analysis of all samples (11 replicates in total from 4 conditions) based on gene expression data (transcripts per kilobase million or TPM) was performed with Python [33]. The Clust tool [34] was used for co-expressed gene clusters identification across all samples. The default normalization procedure of Clust for RNA-seq TPM data (quantile normalization followed by log2-transformation and Z-score normalization, code “101 3 4”) was applied. gProfiler [35] was utilized for the simultaneous functional enrichment analysis of the genes from all clusters in multi-query mode. The RNA-Seq data from this publication have been deposited to the NCBI GEO repository (https://www.ncbi.nlm.nih.gov/geo) and can be accessed with the dataset identifier GSE171853.

### Statistical analysis

Data were analysed using GraphPad Prism (GraphPad Software, La Jolla, CA, USA). Single comparisons were tested using unpaired Student’s *t*-tests for normal distributed samples or Mann–Whitney-U tests when normal distribution was not given. One-way or two-way ANOVA was applied for multiple comparison analysis, with Bonferroni’s multiple comparison post-test. For RT-qPCR unpaired Student’s *t*-test with Welch’s correction was applied. *P* values < 0.05 were estimated as significant (*p < 0.05; **p < 0.005; ***p < 0.0005). Data are presented as mean ± SD.

## Results

### Genetic or pharmacological ISR inhibition sensitizes CML cells to imatinib in vitro and in vivo

**Fig. 1:**
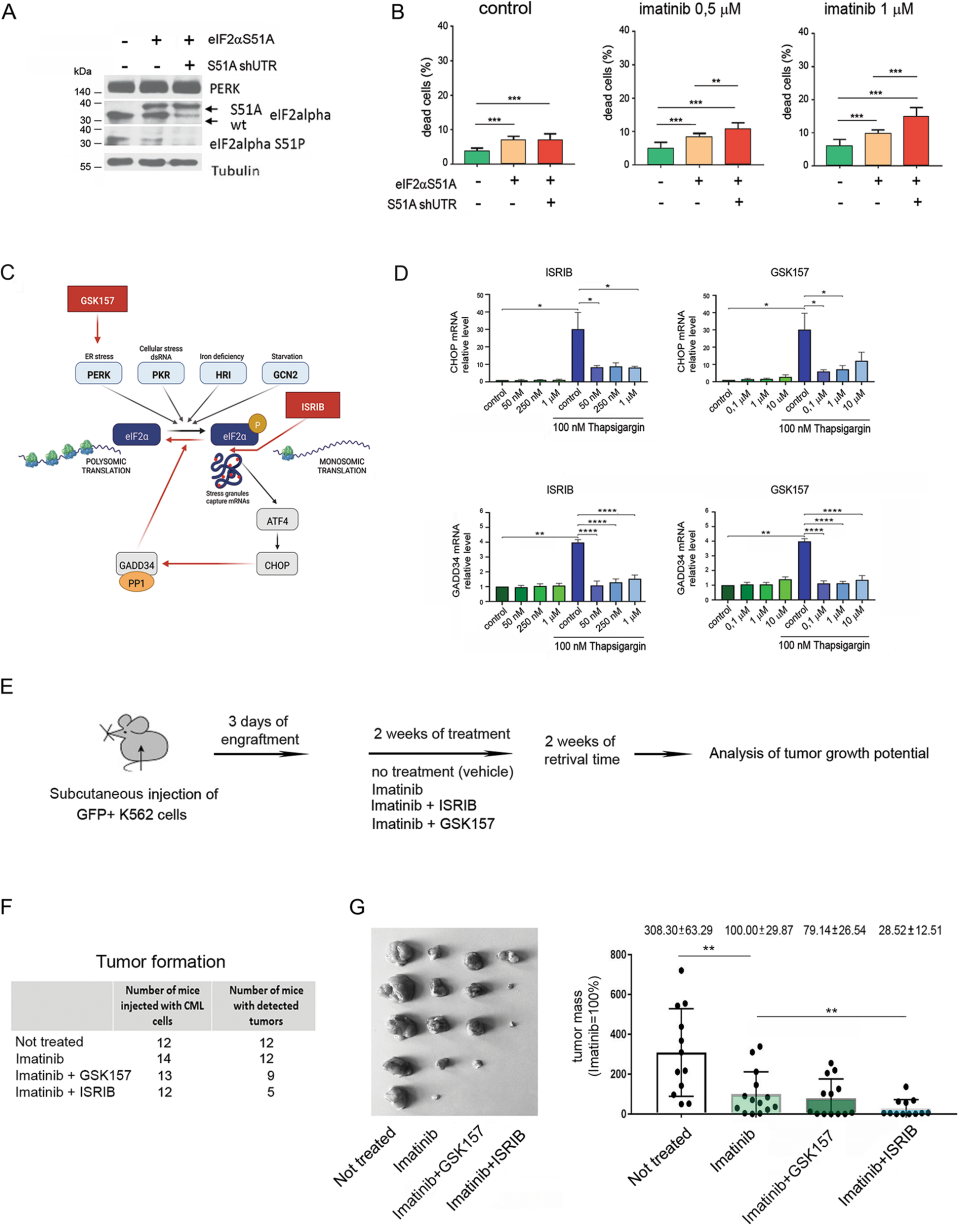
Genetic or pharmacological ISR inhibition sensitizes CML cells to imatinib in vitro and in vivo. **A** The levels of PERK, eIF2α and phosphorylated eIF2α (S51P) protein estimated by western blot in wild-type (wt), or stably transfected eIF2α S51A and S51A shUTR K562 mutants. Arrows indicate wt (lower) and mutated (40 kDa higher) eIF2α bands. Tubulin was used as a loading control. The full-length membranes were properly cut based on the protein marker size according to target protein sites prior to hybridization with primary antibodies (see Materials and Methods). Cropped blots are presented; original non-cropped membranes are shown in the Supplementary Fig. S9. **B** Apoptotic (Annexin V-positive) cells detected by flow cytometry in K562 wt, eIF2αS51A and S51A shUTR mutant cells in untreated conditions or after treatment with 0,5 and 1 μM imatinib. Data are shown as a percentage of dead cells. Statistical analysis: Unpaired Student’s *t*-test with Welch’s correction (*p ≤ 0.05; **p ≤ 0.005; *** *p* ≤ 0.0005). **C** Schematic graph of the integrated stress response (ISR) signaling pathway with the site of ISR inhibitors - ISRIB and GSK157 action (PKR – Protein Kinase R, PERK – PKR-like ER kinase, HRI – Heme-regulated eIF2α kinase, GCN2 – General Control Non-depressible protein 2, eIF2α – eukaryotic translation initiation factor-2α, ATF4 – Activating Transcription Factor 4, CHOP – CCAAT/enhancer-binding protein (C/EBP) homologous protein, GADD34 – Growth Arrest and DNA damage-inducible protein 34, PP1 – protein phosphatase 1). **D** CHOP and GADD34 mRNA expression levels measured by RT-qPCR. Wild-type K562 cells were preconditioned with either ISRIB or GSK157 inhibitors in indicated concentrations for 2 hours, followed by ISR induction with 100 nM thapsigargin for 2 hours to mimic ISR in vitro. For mRNA analysis the level of not treated cells (control) was used as a reference =1. Statistical analysis: unpaired Student’s t-test with Welch’s correction and p ≤ 0,05 was estimated as significant (*p ≤ 0.05; **p ≤ 0.005; ***p ≤ 0.001; ****p ≤ 0.0005). **E** The workflow of the in vivo Xenograft experiment. Mice were: not treated (*n* = 12); or treated with: imatinib (*n* = 14); imatinib and GSK157 (*n* = 13); imatinib and ISRIB (n = 12). **F** Left panel - the number of mice which developed tumors upon all tested conditions. Right panel – pictures of tumors isolated form representative experiment (*n* = 5 mice for each condition). **G** The tumor mass indicating viability and proliferation potential isolated from mice in indicated variants. Tumors grown in mice injected with K562 cells and treated with imatinib were used as a control = 100%. Statistical analysis: Unpaired Student’s *t*-test, F-test to compare variances (*p ≤ 0.05; **p ≤ 0.005)

To study the impact of the ISR, it was first inhibited by genetically targeting its main regulatory hub, eIF2α (eukaryotic initiation translation factor 2α), by expressing the nonphosphorylable form of eIF2α, visible as an additional band on western blotting (obtained cell line: S51A), followed by overexpression of shRNA against the eIF2α 3’UTR (S51A shUTR) to inhibit the expression of endogenous wt eIF2α, leading to a complete lack of eIF2α phosphorylation (Fig. 1A, detailed procedure for the generation of genetically modified cells is provided in the Supplementary Information). Both generated cell lines had unaffected levels of PERK, a UPR kinase acting upstream of eIF2α, and expressed GFP necessary for FACS sorting (Fig. 1A, Fig. S1A). Decreased expression of mRNAs encoding the ISR markers *CHOP* and *GADD34* confirmed that inhibition of eIF2α phosphorylation attenuates the dynamics of ISR activation in leukemic cells (Fig. S1B). We found that inhibition of the ISR by genetic targeting of eIF2α phosphorylation decreased viability and induced apoptosis of K562 CML cells (Fig. 1B). Furthermore, ISR attenuation sensitized cells to imatinib, confirmed by higher apoptosis detected by Annexin V staining in S51A cells and a further increase in S51A shUTR cells compared to wt cells (Fig. 1B). This implies that K562 cells utilize the eIF2α phosphorylation-dependent mechanism to survive and that ISR inhibition sensitizes CML cells to imatinib. Notably, genetic tools target only one regulatory molecule of the ISR signaling network; therefore, these data should be interpreted as a proxy, not fully resembling multidimensional changes in signaling occurring in CML development.

Therefore, as a next step, we verified pharmacological targeting of ISR activated in wild-type K562 cells, and tested two ISR inhibitors: GSK2656157 (GSK157) and ISRIB (Fig. 1C). GSK157 (compound based on heteroaryl acetamide analogue) is an ATP-competitive cell-permeable inhibitor of PERK which binds a kinase ATP pocket with an IC50 of 0.9 nmol/L and stops PERK-dependent ISR activation [24, 36]. The small molecule ISRIB blocks eIF2α-P-dependent downstream signaling with IC50 of 5 nM and inhibits the executive part of the ISR without cytotoxic effects in vivo [37–40]. Cryo-EM studies showed that ISRIB stabilizes eIF2B by binding its subunits and stapling two halves of the protein complex together, therefore reverses the attenuation of eIF2B by phosphorylated eIF2α and attenuates translation [41, 42].

Neither drug has been tested in chronic myeloid leukemia, and only very limited studies are available for the use of these drugs in other myeloid neoplasms. Preconditioning with increasing concentrations of either GSK157 or ISRIB together with ISR induction by the model agent thapsigargin, commonly used in vitro*,* to mimic strong physiological ISR induction [43–45] (Fig. S1C) revealed that both ISR inhibitors significantly reduced the expression of ISR markers at the mRNA levels at the lowest analyzed doses, therefore confirming their efficiency (Fig. 1D).

The results obtained in vitro (Fig. 1A, B) imply that ISR inhibitors might improve imatinib efficacy and eliminate CML cells. To test this hypothesis and verify cell survival and growth potential in vivo, we performed xenograft studies using NSG immunodeficient mice, which were injected with wild-type K562 GFP+ cells and subjected to treatment (experimental design - Fig. 1E). After 2 weeks of treatment with imatinib alone or in combination with ISR inhibitors followed by 2 weeks of retrieval, we observed that all untreated mice developed tumors (100%), whereas the combination of imatinib and ISRIB significantly decreased the number of animals with tumors, with only 5 out of 12 injected mice developing tumors (41%) (Fig. 1F). An example of tumors developed in one representative experiment with 5 mice in each group is shown in the right panel of Fig. 1F. This was associated with significantly decreased tumor mass, which is a function of growth, cell death and proliferation potential of leukemic cells, indicating significantly greater eradication of leukemic cells upon imatinib and ISRIB combination treatment (Fig. 1G) than upon imatinib treatment alone. Conversely, imatinib combined with GSK157, compared to imatinib alone, exerted only a moderate inhibitory effect, and the average tumor mass was not significantly different between those two conditions. These results show that inhibition of the ISR by ISRIB but not GSK157 sensitizes CML cells to imatinib in vivo.

### Combination of imatinib and ISRIB specifically inhibits STAT5 signaling in BCR-ABL1-expressing CML cells

Previous data indicated that the combination of imatinib and ISRIB specifically eliminates BCR-ABL1-expressing cells. To investigate the possible mechanism of the combined treatment (imatinib+ISRIB) compared to treatment with single agents, we verified the activation of signaling pathways that are common drivers of imatinib/TKI resistance in CML [5, 8, 9]. Therefore, two CML cell lines, K562 and LAMA-84, were used to assess the phosphorylation of STAT5 and ERK (effector kinase of the RAS/RAF/MAPK pathway) and members of the AKT/mTOR/S6 and GSK3β signaling pathways in response to imatinib alone, ISRIB alone and imatinib combined with ISRIB. As culturing cell lines under in vitro conditions optimized to provide the best settings for proliferation and survival leads to only very low activation of the ISR, to mimic the physiologically stronger ISR activation (similar to the higher activation observed in vivo in CML patients) in vitro, low-dose (nontoxic), 100 nM thapsigargin was used (Fig. S1C). This is a commonly used treatment and allowed us to test the proposed drug combination in a physiology-mimicking ISR signaling system.

First, we confirmed that the combination of imatinib and ISRIB inhibited the effector part of the ISR, as visualized by a significant decrease in ATF4 levels (Fig. S2A). Due to the site of ISRIB action, phosphorylation of the upstream ISR element eIF2α was not attenuated. ISRIB treatment alone led to a very slight decrease in ATF4, and similar insignificant effects were also observed after imatinib treatment alone. This finding indicated the regulatory link between BCR-ABL1-mediated signaling and ISR induction, confirming the necessity for the combined treatment. In contrast, in K562 cells without visible ISR-ATF4 activation in vitro (without thapsigargin), no significant changes were found (Fig. S2A). However, untreated leukemic cells showed a very low but visible level of ATF4, which was inhibited to undetectable levels upon imatinib treatment alone or imatinib combined with ISRIB treatment (Fig. S2A). This again suggests the existence of moderate, internal stress signaling mediated by oncogenic BCR–ABL1 activity itself, leading to low ISR induction.

**Fig. 2:**
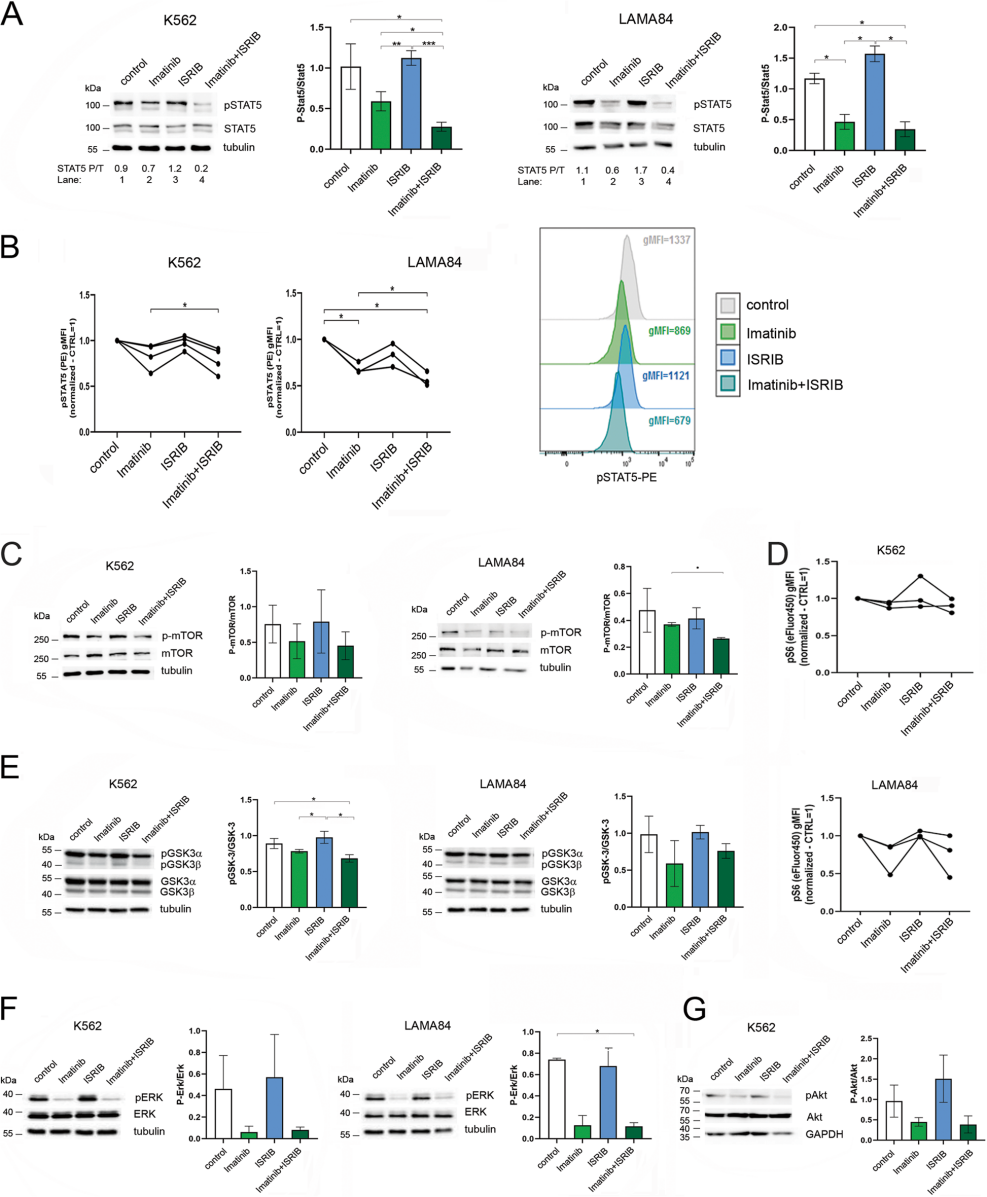
Combination of imatinib and ISRIB specifically inhibits STAT5 signaling in BCR-ABL1-expressing CML cells. **A** Protein levels of STAT5 and phosphorylated form of STAT5 (pSTAT5) detected by western blot in K562 or LAMA84 CML cells. If indicated (ISRIB alone and imatinib+ISRIB conditions), 250 nM ISRIB was added for 2 hours to protect from ISR. 100 nM thapsigargin was added for 2 hours treatment to all experimental conditions to mimic activation of ISR in vitro. This was followed by treatment with 1 μM imatinib, if indicated (imatinib alone and imatinib+ISRIB conditions). After 16 hours cells were collected for analyses. The ratio of phosphorylated to total STAT5 forms (P/T) calculated based on the densitometry signal is given for each condition. Adequate graphs showing pSTAT5/STAT5 ratios are presented. Statistical analysis: unpaired Student’s *t*-test with Welch’s correction (*p ≤ 0.05; **p ≤ 0.005; ***p ≤ 0.0005). Only comparisons with statistical significance are marked. **B** Flow cytometry analysis of pSTAT5 levels in K562 and LAMA84 cells untreated (control) or treated as indicated above. Data were calculated based on gMFI, fluorescence signal for untreated cells = 1. Statistical analysis: repeated-measures one-way ANOVA with Tukey’s multiple comparisons test (*p ≤ 0.05). Right panel - overlay of the representative histograms presenting fluorescence signals for pSTAT5 estimated in control cells or in cells after treatment. gMFI values are indicated for each condition. Only comparisons with statistical significance are marked. **C** Protein levels of mTOR and phosphorylated form of mTOR (p-mTOR) detected by western blot in K562 or LAMA84 CML cells untreated (control) or treated with drugs as indicated above. Adequate graphs showing p-mTOR/mTOR ratios in K562 cells and LAMA84 CML cells are presented. Control cells (without drug treatment)=1. Statistical analysis: unpaired Student’s *t*-test with Welch’s correction (*p ≤ 0.05; **p ≤ 0.005; ***p ≤ 0.0005). Only comparisons with statistical significance are marked. **D** Flow cytometry analysis of pS6 levels in K562 and LAMA84 CML cells untreated (control) or treated as indicated. Data are calculated based on gMFI, fluorescence signal for untreated cells = 1. Statistical analysis: repeated-measures one-way ANOVA with Tukey’s multiple comparisons test (*p ≤ 0.05). Only comparisons with statistical significance are marked. **E**, **F**, **G** Protein levels of GSK3β and phosphorylated form of GSK3β (pGSK3b) (**E**), ERK and phosphorylated form of ERK (pERK) (**F**) and AKT and phosphorylated form of AKT (pAKT) (**G**) detected by western blot in K562 or LAMA84 CML cells untreated (control) or treated with drugs as indicated above. Adequate graphs showing phospho/total protein ratios are presented. Statistical analysis: unpaired Student’s *t*-test with Welch’s correction (*p ≤ 0.05; **p ≤ 0.005; ***p ≤ 0.0005). Only comparisons with statistical significance are marked. The full-length membranes were adequately cut based on the protein marker size according to target protein sites prior to hybridization with primary antibodies (see Materials and Methods). Cropped blots are presented; original non-cropped membranes are shown in the Supplementary Fig. S9

We found that the combination of imatinib and ISRIB specifically decreased STAT5 phosphorylation in both model cell lines in which ISR was active (+TG), which was detected by western blot (Fig. 2A) and confirmed by phospho-flow cytometry (Fig. 2B). ISRIB alone had no effect on STAT5 phosphorylation, whereas imatinib alone decreased it but less efficiently than the double treatment. Notably, both cell types responded slightly differently to imatinib treatment alone (in K562 cells, it was almost not effective, whereas a stronger inhibitory effect on phosphorylated STAT5 was visible in LAMA84 cells); nevertheless, the drug combination was significantly more effective in both cell lines. In contrast, in cells without activated ISR (−TG), imatinib alone was able to inhibit STAT5 signaling (Fig. S2B). Such STAT5 inhibition was also visible upon combined treatment but not after ISRIB treatment alone. This confirms that active ISR mediates, in addition to BCR-ABL1, the active state of STAT5 signaling. Therefore imatinib alone is able to efficiently inhibit STAT5 phoshorylation only when ISR signaling is not active.

We also detected attenuation of the ERK and AKT/mTOR pathways (estimated by the phosphorylation levels of ERK, AKT, mTOR and S6) by imatinib+ISRIB treatment; this effect was observed after imatinib treatment alone and upon the combination treatment of imatinib and ISRIB (Fig. 2C, D, F, G). Similar effects were found in cells without thapsigargin treatment, indicating that ERK and AKT signaling are not regulated or activated by the ISR. (Fig. S2B). Nevertheless, it does not exclude the possible additive inhibitory effect of the drug combination in cells in which imatinib alone would not be effective due to other reasons. On the other hand, we did not observe decreased GSK3β phosphorylation in response to any of the treatment conditions, either in cells without or with ISR activation (Fig. 2E, Fig. S2B). The cell lines used here typically lack BCR-ABL1 mutation, and inhibition of BCR-ABL1 activity but not a decrease in BCR-ABL1 protein levels was observed after treatment with imatinib alone and imatinib in combination with ISRIB (Fig. S3A, B). Additionally, by measuring the level of cell death by flow cytometry, we ruled out possibility that the signaling and mechanistic changes observed in the phosphorylation levels of the regulatory proteins, result from cell death (Fig. S3C). Altogether, these results showed that the ISR, in addition to BCR-ABL1, is responsible for maintaining STAT5 in an active state and preventing its inhibition by imatinib. The obtained data support our observation that the combination of imatinib and ISRIB has a substantial effect, specifically inhibiting proleukemic STAT5 signaling in CML cells.

### Imatinib combined with ISRIB attenuates engraftment of primary TKI-refractory CML CD34+ blasts

The results in Figs. 1 and 2 imply that the combination of imatinib and ISRIB specifically inhibits STAT5 but possibly also the AKT and ERK signaling pathways, which are described as resistance drivers, to sensitize cells to imatinib. We propose that such combined therapy might be efficient in eliminating TKI-resistant primary CML cells with hyperactivated STAT5, often leading to BCR-ABL1-independent resistance. We verified this hypothesis in vivo in a PDX model using immunodeficient NSG mice as hosts bearing primary CD34+ CML cells resistant to imatinib and dasatinib and carrying a pathogenic variant of a gain-of-function mutation in the *PTPN11* gene (Gly60Val/c.179G > T) but not mutations within the kinase domain of BCR-ABL1, as identified by NGS analysis of samples from the clinical resistance time point. *PTPN11* (*SHP2*) gain-of-function mutations block autoregulation of SHP2 catalytic activity, leading to uncontrolled hyperactivation of the RAS/RAF/MAPK and JAK/STAT5 pathways, resistance and poor overall survival, which have also been identified in myeloid leukemia [46, 47]. Therefore, they represent a BCR-ABL1-independent, imatinib/TKI resistant phenotype based on ERK and STAT5 overactivation. The detailed patient characteristics are provided in the Supplementary Information.

**Fig. 3:**
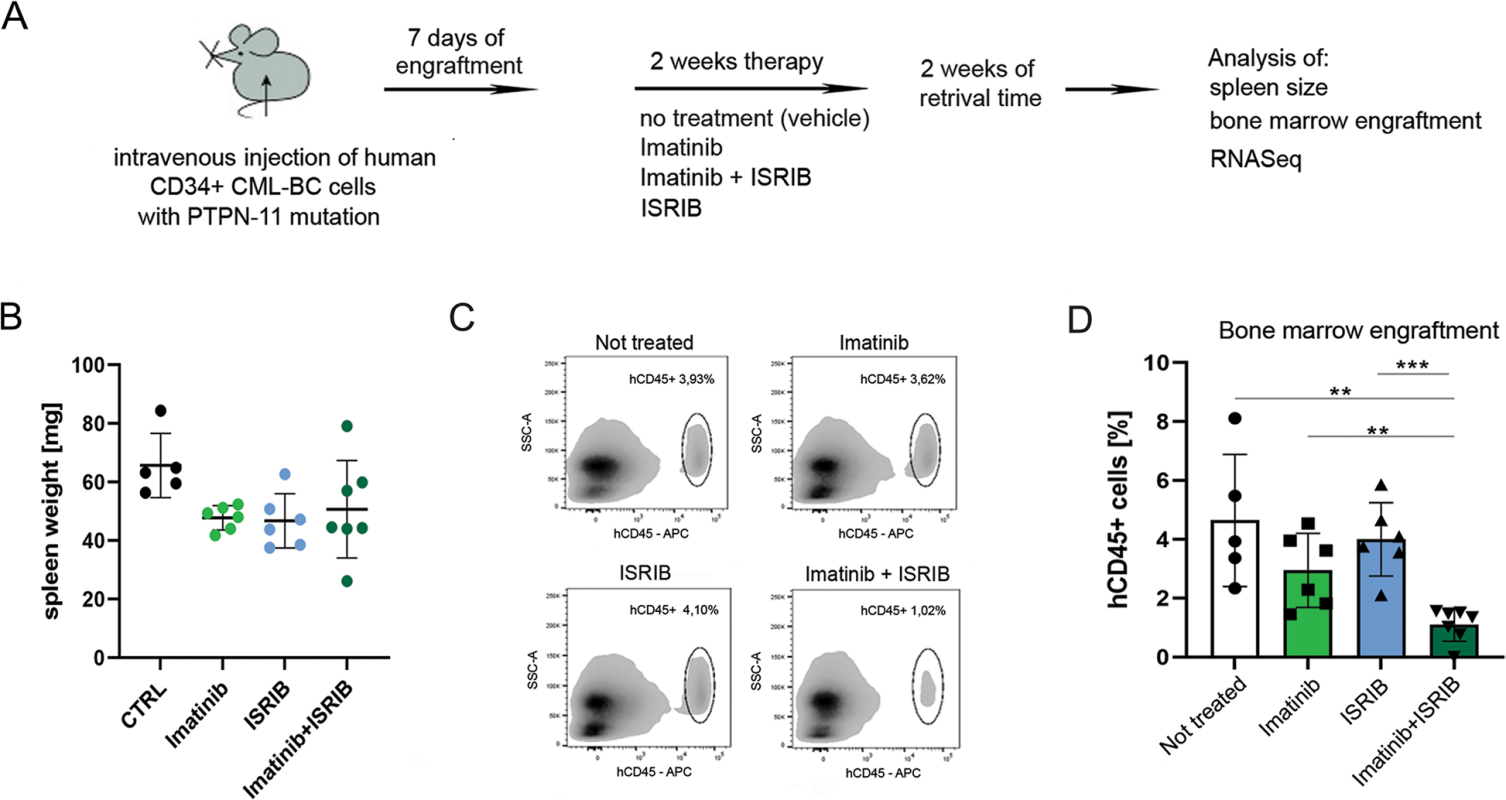
Imatinib combined with ISRIB attenuates engraftment of primary TKI-refractory CML CD34+ blasts. **A** The workflow of the in vivo experiment. Patient-Derived Xenograft (PDX) mice were: not treated/vehicle administrated (n = 5); or treated with: imatinib (*n* = 6); ISRIB (*n* = 7); or combination of imatinib and ISRIB (n = 7). **B** Weight of spleens isolated from mice not treated or treated as indicated. **C** Representative density plots showing the engraftment of hCD45+ CML primary cells into the bone marrow population after the therapeutic treatment, detected by flow cytometry. hCD45+ population is gated on the hCD45 vs SSC dot plots, the percentage of hCD45+ cells within bone marrow (BM) aspirates is indicated. **D** Corresponding graph showing the bone marrow engraftment estimated by flow cytometric detection of hCD45+ CML primary cells in bone marrow aspirates, in given variants of treatment. The percentage of hCD45+ cells is shown. Statistical analysis: Unpaired Student’s *t*-test, F test to compare variances (*p ≤ 0.05; **p ≤ 0.005; ***p ≤ 0.0005). Only comparisons with statistical significance are marked

A short and aggressive 7-day engraftment regimen was applied in the PDX model to test the beneficial effects of treatment with imatinib or ISRIB alone or with the drug combination (experimental scheme and treatment - Fig. 3A). Briefly, after the engraftment time, the animals were treated for 14 days, followed by 2 weeks of retrieval. All variants of treatment showed noticeable but not significant decreases in spleen weight (Fig. 3B). To estimate the short-term engraftment, the percentage of human CD45+ (hCD45+) cells was detected within the bone marrow population using flow cytometry (Fig. 3C, D; the gating strategy for flow cytometry - Fig. S4). We observed that the combination of imatinib and ISRIB significantly attenuated engraftment into the bone marrow, visible as a decreased percentage of hCD45+ cells within the bone marrow population, with a 2- to 3-fold lower level than those of treatment with imatinib or ISRIB alone. In contrast, treatment with imatinib alone did not decrease CML engraftment into the bone marrow. Moreover, to check the nature of drug interaction, the CDI (coefficient of drug interaction) was calculated. The CDI of the drug combination was 0.32, what indicated significant synergism (CDI < 1 indicates synergism, CDI < 0.7 indicates a significant synergistic effect) (Fig. 3D). Altogether, we showed that the combined treatment eradicates resistant CML blasts and decreases leukemia engraftment, therefore indicating the possible synergistic effect of ISRIB and imatinib.

### Combination of Imatinib and ISRIB reprogrammes the gene expression profile of primary TKI-resistant blasts

**Fig. 4:**
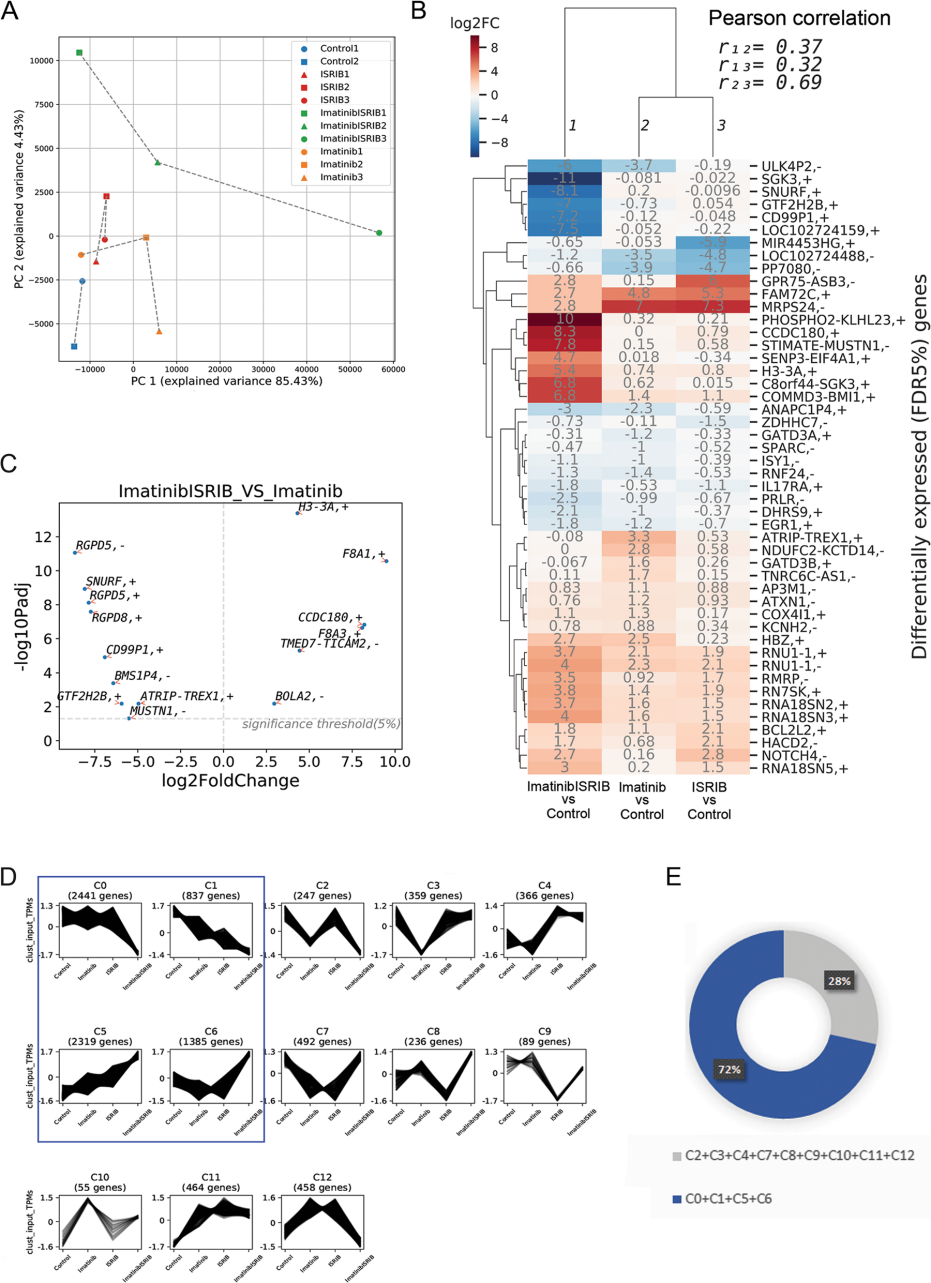
Combination of imatinib and ISRIB reprogrammes the gene expression profile of primary TKI-resistant blasts. **A** Two-dimensional principal component analysis plot of samples based on gene expression (TPM) data obtained from FACS-sorted hCD45+ CML cells isolated from untreated control mice (*n* = 2, blue), or treated with ISRIB (*n* = 3, red), imatinib alone (n = 3, orange) or with combination of imatinib and ISRIB (n = 3, green). **B** Hierarchically clustered heatmap of fold-changes in expression (log2FoldChange) of significantly differentially expressed genes between the indicated pairs of conditions. Pairwise correlations of expression fold-changes are also shown. **C** Significantly altered genes upregulated (positive value on x-axis) or downregulated (negative value on x-axis) in combined imatinib and ISRIB treatment versus with imatinib alone. **D** Clusters (C0-C12) of co-expressed genes with varying patterns of gene expressions across all variants of treatment. Clusters C0, C1 displaying sharp downregulation or C5, C6 showing sharp upregulation of gene expression after combined treatment are marked in blue frame. **E** Diagram showing the percentage of genes identified in four selected clusters C0, C1, C5, C6 (blue) and the rest (grey). Transcriptome analysis has been done on hCD45+ cells isolated from BM respirates of PDX mouse model with CD34+ CML-BP imatinib resistant blasts carrying *PTPN11 *mutation

To investigate the molecular effects of the double treatment, RNA-seq was performed on FACS-sorted hCD45+ CML cells isolated from the bone marrow of PDX mice bearing CD34+ *PTPN11*-mutated CML cells and treated as described above. Principal component analysis (PCA) indicated that cells treated with imatinib and ISRIB are transcriptionally distinct (Fig. 4A). This was confirmed by hierarchical clustering of significantly changed genes between pairs of tested conditions (treatment vs. control) and supported by the Pearson correlation values, which showed a higher correlation between sole ISRIB and sole imatinib treatments (both compared to control) (r = 0.69) than between each of the single treatments and the combined imatinib+ISRIB treatment (all compared to control) (r = 0.32 and 0.37, respectively; Fig. 4B). The *SGK3* and *SNURF/SNRPN* genes regulating alternative RNA processing were identified as significantly downregulated upon double treatment. Most upregulated genes encoded proteins regulating transcription and RNA processing. To identify genes responsible for the increased sensitivity appearing after the double treatment, the gene expression profiles for imatinib versus imatinib+ISRIB were compared. In addition to the previously described genes (Fig. 4B), genes encoding proteins from the small GTP-binding RAS superfamily (*RGPD5* and *RGPD8*) were significantly downregulated (Fig. 4C, data for all treatment combinations see Fig. S5A).

Next, to evaluate remodeling of gene transcription more globally and recognize patterns of gene expression, clusters (C0-C12) of coexpressed genes across all variants of treatment were identified (all detected genes included) (Fig. 4D). Clusters with the highest number of genes represented the groups in which drug combination led to either a sharp decrease (C0, C1) or increase (C5, C6) in gene expression (Fig. 4D, E). These four clusters included approximately 72% of all detected genes (Fig. 4E). This further confirms that the gene expression pattern for the imatinib+ISRIB combination is specific and different from the other treatment conditions.

### Combination of imatinib and ISRIB downregulates genes related to RAS/RAF/MAPK and STAT5 signaling

**Fig. 5:**
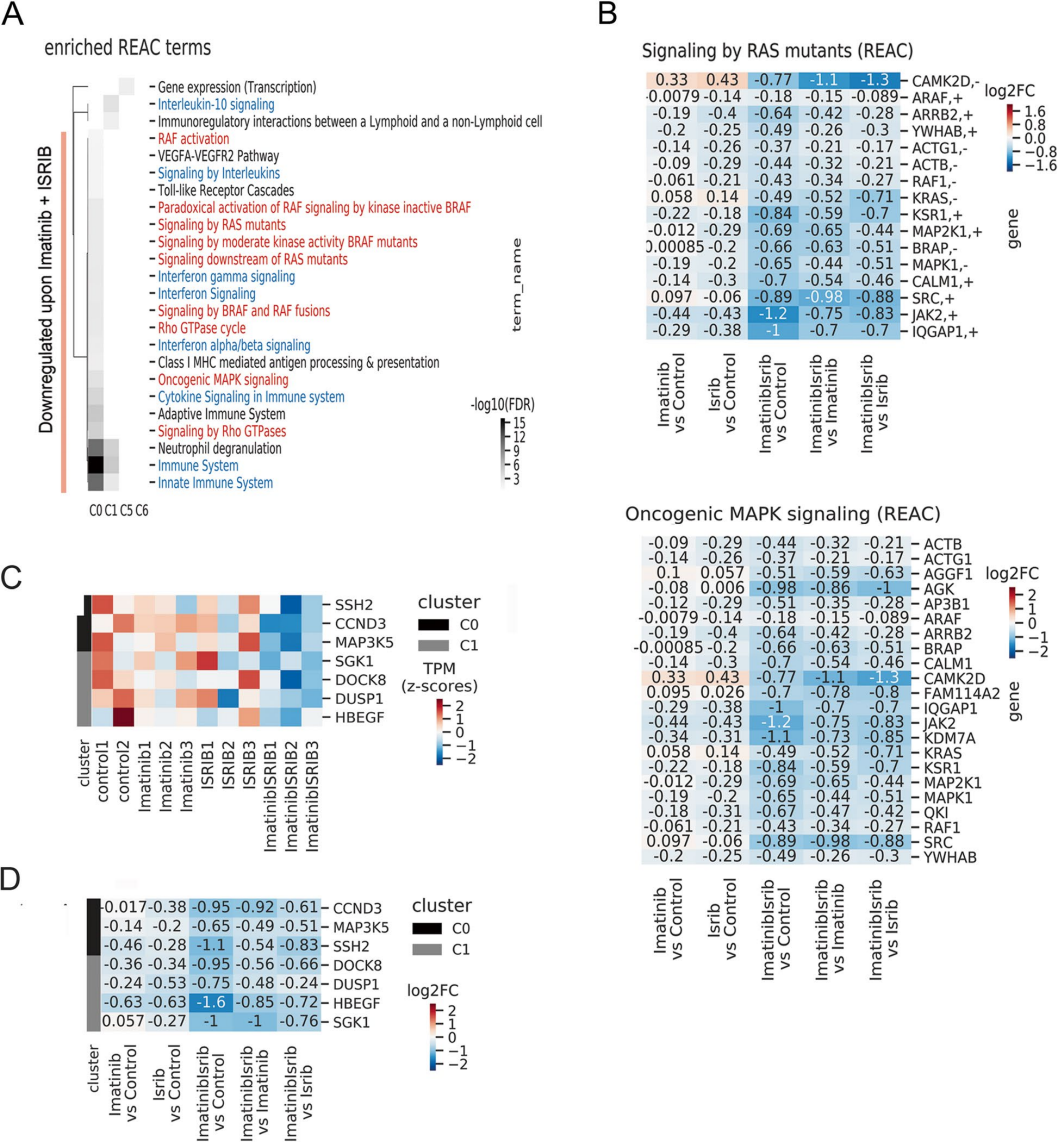
Combination of imatinib and ISRIB downregulates genes related to RAS/RAF/MAPK and STAT5 signaling. **A **Functional enrichment Reactome (REAC) [48] terms significantly enriched in C0, C1, C5 and C6 clusters. Downregulated genes belonging to C0, C1 clusters are indicated. RAS signaling is marked in red color, Interferon gamma signaling is marked in blue color. **B **Heat map showing changes in expression levels (Log2FoldChange) of selected genes related to RAS (upper) and MAPK (lower) signaling for indicated pairs of conditions. **C** The heat map showing expression level (transcript per kilobase million or TPM, standardized with z-score) of STAT5-target genes belonging to C0, C1 clusters shown for each gene across all replicates of untreated (control) and treatment conditions. **D** The change in expression of STAT5-target genes belonging to cluster C0 and C1 in treatments comparison: expression fold change (log2FoldChange) in all comparisons. Transcriptome analysis has been done on hCD45+ cells isolated form BM respirates of PDX mouse model with CD34+ CML-BP imatinib resistant blasts carrying *PTPN11 *mutation

To predict the cellular mechanisms altered by the combined treatment, all 13 defined gene clusters underwent functional enrichment analysis. The C0 and C1 clusters, which included genes downregulated upon the combined treatment, were significantly enriched in terms related to RAS/RAF/MAPK signaling (Fig. 5A, marked in red; for all clusters, see Fig. S5B). Specifically, for RAS and MAPK signaling, the genes encoding RAF1, ARAF, ERK2, KRAS, SRC, JAK2 and a number of proteins involved in the activation of the MAPK cascade, such as MEK1, MAPK1, MAP4K1, MAP3K3, MADD, were downregulated upon imatinib+ISRIB treatment compared to the control or both single treatments (Fig. 5B). The imatinib+ISRIB drug combination also attenuated IFNγ signaling and the immune response, which in leukemia can additionally mediate activation of the JAK2/STAT5 pathway and the inflammatory response (Fig. 5A, marked in blue; for all clusters, see Fig. S5B). Downregulation of processes essential for the leukemia-promoting kinase-dependent signaling and immune response was also confirmed by the Gene Ontology biological process (BP) terms that were enriched (Fig. S6, see C0 and C1 clusters).

Even if the *SGK3* gene that encodes the serine/threonine-protein kinase SGK3 was significantly downregulated after the combined treatment (Fig. 4B), the effect on the SGK3 signaling pathway was excluded based on the analysis of the phosphorylated levels of SGK3 as well as the expression of the SGK3 interaction partners selected based on the interaction partner data source: BioGRID, IntAct (EMBL-EBI) and APID databases (see Supplementary Information) (Fig. S7).

Altogether, these results showed that genes related to RAS/RAF/MAPK and STAT5 oncogenic pro-leukemic signaling were downregulated upon the combination treatment of imatinib and ISRIB, presumably enhancing the targeting of leukemia cells by imatinib.

Transcriptomic data showing that the combined treatment can downregulate oncogenic RAS/RAF/MAPK, JAK2, and IFNγ signaling as well as genes that are mediators of JAK2/STAT5 signaling (Fig. 5, Fig. S5B, Fig. S6) correlated with decreased phosphorylation of AKT, ERK and STAT5 proteins verified by western blotting (Fig. 2). Therefore, to confirm the specific downregulation of STAT5-dependent downstream pathways, fold change analysis of STAT5 target genes was performed on the genes downregulated upon imatinib+ISRIB treatment. The list of possible STAT5 target genes was created based on ChIP-Seq data from malignant hematopoietic cells (for details, see Supplementary Information). We found that the combined treatment decreased the expression of STAT5 target genes (*SSH2, CCND3, MAP 3 K5, SGK1, DOCK8, DUSP1 and HBEGF*) compared to the control and single treatments (Fig. 5C, D). The downregulated STAT5 target genes encoded positive regulators of cell cycle/proliferation, stress response and survival, including slingshot protein phosphatase, cyclin D3, ZIR8, MAP kinase phosphatase 1, EGF-like growth factor, MAP3K5 and SGK1. Data for all clusters are presented in Fig. S8. Conversely, this inhibitory effect was not observed for imatinib or ISRIB treatment alone.

Altogether, we discovered that inhibition of the ISR by the small molecule ISRIB combined with imatinib is highly effective, sensitizes cells to imatinib and eliminates CML blasts. Moreover, this drug combination attenuates cytoprotective RAS/RAF/MAPK/ERK and STAT5 signaling in CML-BC cells, and correlates with decreased expression of STAT5 target genes related to proliferation and cytoprotection. Therefore, we propose a novel treatment strategy based on the combination of imatinib and ISRIB, which can be considered an effective therapy to eradicate leukemic cells showing resistance due to hyperactivation of the RAS/RAF/MAPK and STAT5 signaling pathways.

## Discussion

The development of imatinib has revolutionized CML treatment and overall patient survival. Despite the clinical success of imatinib in the chronic phase of treatment, the disease is still not fully curable, and eradication of all leukemic cells has not been efficiently achieved. Imatinib intolerance or primary resistance occurs, and many patients develop secondary resistance due to activation of intrinsic signaling pathways, including the JAK/STAT5, GSK3β and RAS/RAF/MAPK/ERK pathways [5, 6, 8, 9]. Importantly, such activation usually occurs in a BCR-ABL1-independent manner; thus, even upon imatinib treatment of leukemic cells, including BCR-ABL1 nonmutated cells, those oncogenic pathways remain active.

Here, we provide evidence that inhibition of the integrated stress response by ISRIB, combined with the tyrosine kinase inhibitor imatinib, might target both stress response adaptive signaling and RAS/RAF/MAPK/ERK and STAT5-dependent intrinsic prosurvival cytoprotective signaling. This results in the effective elimination of CML cells that are resistant to TKIs due to internal signaling.

Our findings are in line with the currently proposed strategy to eradicate leukemic blasts, which aims to target BCR-ABL1 and simultaneously inhibit oncogenic signaling pathways to resensitize cells to TKIs when resistance is not mediated by BCR-ABL1 mutations [4, 49–51]. This is consistent with the observation by others that chemotherapy combined with ISRIB abrogated breast cancer plasticity and improved therapeutic efficacy [20]. Studies of the clinical potential of ISRIB in hematological malignancies are still limited [52, 53], and this work shows the effectiveness of ISRIB in a combination treatment against CML-BP TKI-resistant cells.

The antileukemic effects of the double treatment were more visible and effective in vivo upon treatment with pharmacological inhibitors than in vitro in genetically modified cells. This might be related to the fact that the physiological ISR signaling network can only be fully activated in vivo by either intrinsic or extrinsic mechanisms, therefore allowing for full effectiveness of drug treatment. In contrast, optimal in vitro conditions in which cell lines are cultured lead to only moderate and low levels of ISR activity. Additionally, we observed that only the small molecule ISRIB, but not another ISR inhibitor, GSK157, belonging to the PERK inhibitor family, was effective in vivo. This is consistent with recent studies of amyotrophic lateral sclerosis that showed data similar to ours [54]. The lower effectivity of GSK157 in vivo can be a result of eIF2α phosphorylation-independent effects [55], moderate specificity of GSK157, as its affinity for RIPK1 was shown to be significantly higher than that of PERK kinase [56], and pancreatic toxicity reported recently [57]. Moreover, as PERK inhibitors precisely target only one of four ISR signaling arms, the possibility that another parallel signaling pathway leading to the ISR is still active in vivo cannot be ignored. The ISRIB molecule, which is a very promising drug intensively investigated in malignant brain conditions and age-related memory decline [58, 59], as well as in some metastatic tumors [16, 42–44, 60, 61], in contrast to PERK inhibitors, acts downstream of phosphorylated eIF2α and directly inhibits the executive part of the ISR [37, 42, 44]. In addition, ISRIB may have other targets that have not yet been identified. The results obtained upon in vivo treatment and presented here provide several possible signaling pathways that may be altered by ISRIB in malignant cells, increasing its effectiveness.

**Fig. 6:**
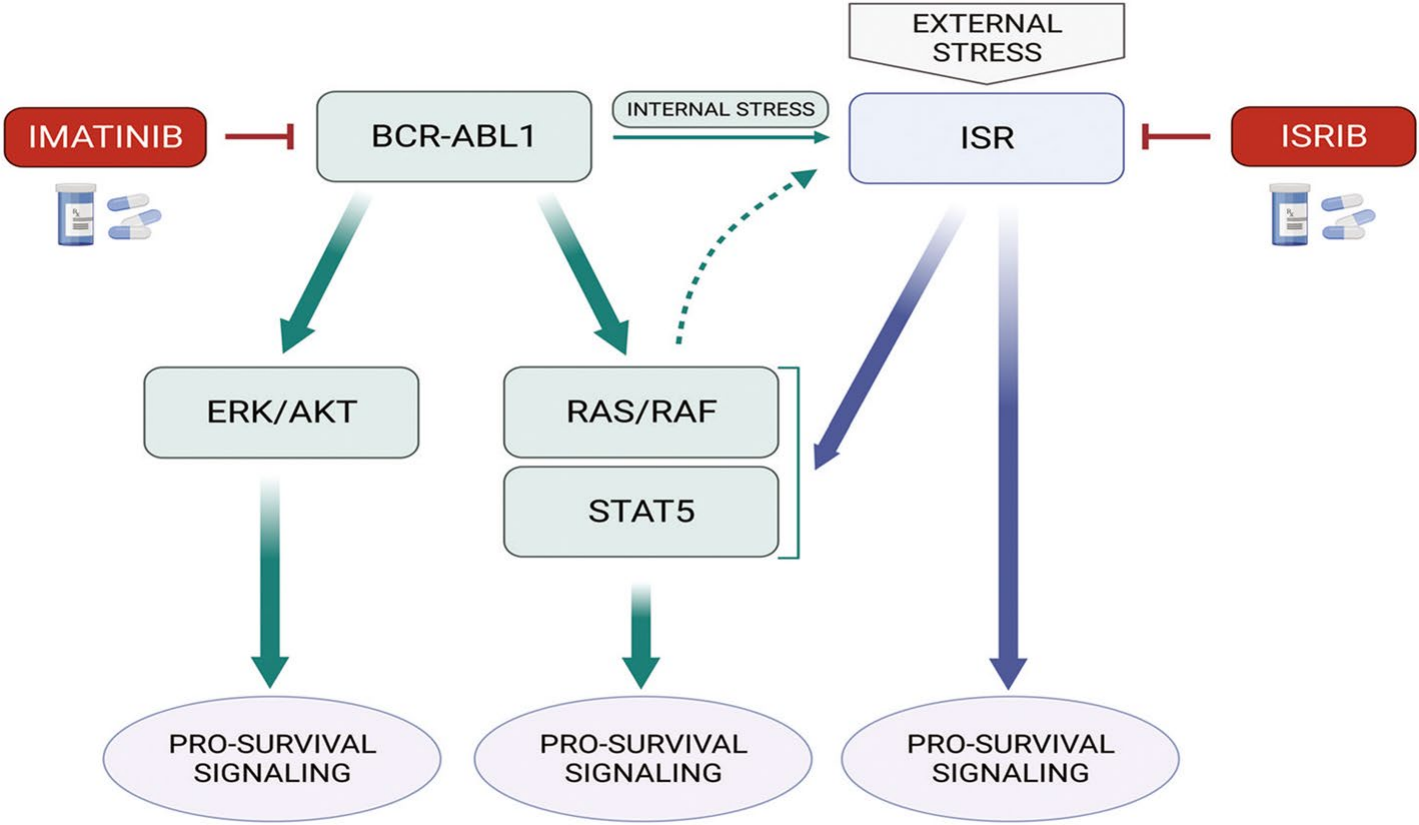
Scheme of the mechanisms targeted by imatinib and ISRIB combination

Mechanistically, we present here that imatinib combined with ISRIB specifically inhibits STAT5 as well as RAS/RAF/MAPK/ERK signaling. This correlates with downregulation of STAT5 target genes, related to proliferation, survival and cytoprotection. We also show that the ISR itself, in addition to BCR-ABL1-dependent signaling, is related to active state of STAT5 and prevents its inhibition by imatinib alone. The regulatory link between ER stress response regulator XBP1 and STAT5 has been shown in pre-B ALL [22]. Additionally, XBP1 promoted transcription of IL-3 and phosphorylation of STAT5 in BaF3 hematopoietic cells [62]. Since ATF4 (transcription factor synthesized dependently on eIF2α phosphorylation status) was described as enhancer of the IRE1-XBP1 signaling [63], the regulatory loop linking ISR, ISRIB-regulated eIF2α-ATF4 signaling and STAT5 was confirmed. Therefore, we can not neglect that another ISR or stress-related regulators, which are indirectly inhibited by ISRIB, are involved in the ISR-dependent activation of STAT5. On the other hand, activity of BCR-ABL1 mediated very slight but still visible ISR-ATF4 activity, which can result from microenvironmental changes like ROS production or oncogene-dependent protein overload. This correlates with activation of ISR, presented also by others [21–23]. Altogether this clearly shows presence of the bi-directional interactions between BCR-ABL1 and ISR signaling, both related to STAT5 activation. This strongly supports our hypothesis that the combined targeting of BCR-ABL1 and the ISR is necessary for the effective antileukemic inhibition of STAT5. Such ISR-dependent protective outcomes were not present in the case of AKT and ERK signaling. The scheme of the proposed mechanisms targeted by drug combination is presented in Fig. 6.

Importantly, even though the mechanistic signaling part of this study was in model cell lines, downregulation of AKT and ERK was visible after imatinib treatment alone as well as after the drug combination treatment. Thus, we have provided direct evidence that such treatment can efficiently downregulate RAS/RAF/MAPK/ERK and STAT5 signaling in *PTPN11*-mutated CD34+ CML-BC cells (not carrying BCR-ABL1 mutation) with RAS/RAF/MAPK and STAT5 hyperactivation. Eradication of those highly resistant cells and lower leukemia engraftment indicated the therapeutic relevance of the combined treatment. Therefore, even if only shown in one highly specific and resistant clinical case, these data provide significant translational value supporting the performance of future studies to verify the treatment of resistant patients with RAS/RAF/MAPK and/or STAT5 hyperactivation, which are cases that are difficult to cure and show low overall survival in myeloid leukemias [10, 64]. Our proposed strategy is supported by others, as targeting STAT5 in CML was shown to effectively overcome TKI resistance and eradicate leukemic cells [65–68]. Additionally, the STAT5 target genes we identified, which were inhibited by the double treatment, have been shown to be possible therapeutic targets in hematological malignancies. This includes genetic or pharmacological inhibition of DUSP1, which abrogates intrinsic resistance to TKIs in BCR-ABL1-induced leukemia [69].

Notably, hyperactivation of RAS/RAF/MAPK and STAT5 signaling has also been detected in other myeloid neoplasms, such as non-CML chronic myeloproliferative disorders correlating with JAK2 V617F mutation [70] and Flt3-ITD-positive AML [71], and in all cases it was correlated with the resistant phenotype and overall poor prognosis and survival [64]. Therefore, it is worth considering that the proposed strategy might also be effective in other hematological malignancies with hyperactivation of RAS/RAF/MAPK and STAT5 signaling. In general, our findings indicate the possible synergistic effect of both drugs and support the strategy based on genetic screening (NGS) to identify patients carrying driver mutations (such as *PTPN11*), which are responsible for hyperactivation of the targeted pathways and are possibly sensitive to the proposed treatment.

Interestingly, the differential expression of genes responsible for immune modulation (visible even in the xenograft model, which excludes the involvement of T and B lymphocytes but still encompasses functional myeloid cells) suggests the possible involvement of immune system remodeling in the therapeutic outcome. These data support the idea of targeting the innate immune response or immune checkpoints in myeloid malignancies, including CML [72–75]. Thus, even though experiments were performed in immunodeficient (lacking adaptive, lymphocyte-mediated response) mice, signaling and functional effects related to the innate immune responses (mediated by macrophages) may have been functional, leading to the observed changes. Although this finding is interesting, it has to be verified in subsequent studies using the syngeneic mouse model.

## Conclusions

We discovered a novel strategy to eradicate imatinib-refractory CML blasts based on the therapeutic combination of the ISR inhibitor ISRIB together with the oncogenic tyrosine kinase inhibitor imatinib. We postulate that such a strategy can improve therapeutic outcomes in leukemic patients showing TKI resistance related to the hyperactivation of RAS/RAF/MAPK, STAT5 and stress adaptation signaling, and selected based on genetic identification. A similar approach based on ISRIB combined with a typical chemotherapy agent may also be applied to other hematological malignancies carrying driver mutations, leading to constitutively activated STAT5 and RAS/RAF/MAPK signaling associated with TKI resistance.

## Supplementary Information

**Additional file 1.** 

## Abbreviations

AKT: Protein kinase B
CHOP: C/EBP homologous protein
CML: Chronic myeloid leukemia
CML-CP: Chronic myeloid leukemia – chronic phase
CML-BC: Chronic myeloid leukemia – blastic phase
eIF2α: Eukaryotic translation initiation factor 2α
ERK: Extracellular signal-regulated kinase
GADD34: Growth arrest and DNA damage-inducible protein
GSK3β: Glycogen synthase kinase 3 Beta
ISR: Integrated stress response
ISRIB: Integrated stress response inhibitor
JAK2: Janus kinase 2
MAPK: Mitogen-activated protein kinase
mTOR: Mechanistic target of rapamycin
NGS: Next generation sequencing
NSG: NOD.Cg-Prkdcscid Il2rgtm1Wjl/SzJ
PDX: Patient derived xenograft
PERK: Protein kinase RNA-Like ER kinase
PI3K: Phosphoinositide 3-kinase
PTPN11: Protein tyrosine phosphatase non-receptor type 11
RNA-Seq: RNA-sequencing
S6K: Ribosomal protein S6 kinase beta-1
SHP2: SH2 containing protein tyrosine phosphatase-2
STAT5: Signal transducer and activator of transcription 5
