# Supplementary material for: Targeting integrated stress response with ISRIB combined with imatinib treatment attenuates RAS/RAF/MAPK and STAT5 signaling and eradicates chronic myeloid leukemia cells
Source: BMC Cancer. 2022 Dec 2;22:1254. doi: 10.1186/s12885-022-10289-w (PMC9719211; doi:10.1186/s12885-022-10289-w)

Supplementary Information

Patient clinical characteristics

Patient (male, 53 years) was diagnosed in Dec. 2010 as BP CML (hyperleukocytosis 120G/L, Ph+, monosomy of chromosome 7, BCR-ABL1 p210+, no BCR-ABL1 mutations, 23% of blasts in the blood, splenomegaly – 200 mm). Treatment initiated with imatinib 600 mg/day. CHR after 1 month, PCyR and MMR with 7,7% BCR-ABL1 after 3 months, CCyR and MMR with 1,15% BCR-ABL1 after 9 months. In Dec. 2011, after 12 months of imatinib treatment, disease relapse and diagnosed progression with BCR-ABL1 p210 53% and increased number of Ph+ and monosomy of chromosome 7 in karyotype. NGS analysis additionally revealed pathogenic variant in *PTPN11* gene in the sample from the clinical resistance time point (Gly60Val/c.179G>T). Discontinued treatment with imatinib, received dasatinib 100 mg/day. No response and no decrease in BP symptoms. Treated with polychemotherapy without effects. Patient died in Oct. 2012.

Generation of cells expressing non-phosphorylable form of eIF2α

Inhibition of eIF2α phosphorylation was utilized by lentiviral transduction. Since eIF2α is crucial for cell survival, development of cell line expressing only mutated, non-phosphorylable form of eIF2α was a two-step process. First, a non-phosphorylable eIF2α S51A mutated form was expressed, to generate cells with partial inhibition of eIF2α phosphorylation (named as S51A, visible as additional band at 40 kDa in Western Blot, Fig. 1). In the second step, eIF2α S51A mutant cells additionally overexpressed shRNA sequence against 3'UTR region of eIF2α, which is present in the wt form exclusively, to inhibit expression of endogenous wt eIF2α. This enabled development of stable cell line (named as S51A shUTR) with an almost non-detectable expression of wt eIF2α and sole expression of mutated form, leading altogether to complete inhibition of eIF2α phosphorylation. Obtained cell lines co-expressed GFP protein, used for FACS sorting.

Identification and expression of STAT5 target genes

The list of possible STAT5 target genes was created based on the CHIP-Seq data from malignant/hematopoietic cells (Alvarez and Frank, 2004; Basham et al., 2008; Kanai et al., 2014; Pinz et al., 2016; Theodorou et al., 2013). Fig. 7E presents the heatmap of expression level (transcript per kilobase million or TPM, standardized with z-score) of each gene across all replicates of control (not treated) and treatments for target genes belonging to clusters C0 and C1 in PDX model data.

Identification and expression of SGK3 interaction partners

SGK3 partner genes were identified based on the interaction partner datasource: BioGRID, IntAct (EMBL-EBI) and APID databases. A list of proteins/corresponding genes that interact with SGK3 and was created and their levels were checked in co-expression clusters C0-C12. Supplementary Figure 6 shows the log2FoldChange of SGK3 interaction partner genes across all treatments, with clusters C0 and C1 marked in red and blue, respectively. If any of analysed gene was not a member of any cluster, it is marked as "None" and colored in grey.

Table 1

List of antibodies used for Western Blot.

Epitop	Company / Cat No.	Dilution
**PERK**	Cell Signaling #5683	1:1000
**eIF2α**	Cell Signaling #2103	1:1000
**phospho-eIF2α (S51)**	Cell Signaling #9721	1:500
**phospho-STAT5 (Tyr694)**	Cell Signaling #9356	1:500
**STAT5**	Cell Signaling #9363	1:500
**phospho-mTOR (Ser2448)**	Cell Signaling #2971	1:1000
**mTOR**	Cell Signaling #2972	1:1000
**phospho-Akt (Ser473)**	Cell Signaling #4060	1:1000
**Akt**	Cell Signaling #4691	1:1000
**phospho-ERK1/2 (Thr202/Tyr204)**	Cell Signaling #4370	1:1000
**ERK**	Cell Signaling #4695	1:1000
**phospho-SGK3 (Thr320)**	Cell Signaling #5642	1:500
**SGK3**	Cell Signaling #8156	1:1000
**phospho-GSK3α/β (Ser21/Ser9)**	Cell Signaling #9331	1:1000
**GSK3α/β**	Cell Signaling #5676	1:1000
**c-ABL (Ab-3)**	Merck #OP20	1:1000
**tubulin**	Merck #CP06	1:5000
**GAPDH**	Santa Cruz Biotechnology #MAB374	1:10000
**Grp78**	BD #610978	1:1000
**ATF4**	Proteintech #10835-1-AP	1:1000
**phospho-Tyrosine (P-Tyr-100)**	Cell Signaling #9411	1:2000
**Goat anti-mouse/HRP secondary Ab**	Dako #P0447	1:2000
**Goat anti-rabbit/HRP secondary Ab**	Dako #P0448	1:2000

Table 2

Primers used in RT-qPCR reaction.

Target	Forward	Reverse
**18SrRNA**	5’ GTAACCCGTTGAACCCC 3’	5’ CCATCCAATCGGTAGTAGCG 3’
**Actin**	5’ CATGTACGTTGCTATCCAGGC 3’	5’ CTCCTTAATGTCACGCACGAT 3’
**CHOP**	5’ AGCCAAAATCAGAGCTGGAA 3’	5’ TGGATCAGTCTGGAAAAGCA 3’
**GADD34**	5’ GTAGCCTGATGGGGTGCTT 3’	5’ TGAGGCAGCCGGAGATAC 3’

Primers were synthesized by Oligo.pl (DNA Sequencing and Oligonucleotides Synthesis Service IBB, Warsaw, Poland).

Supplementary Figure 1.
Genetic inhibition of eIF2α phosphorylation attenuates ISR signaling in vitro.

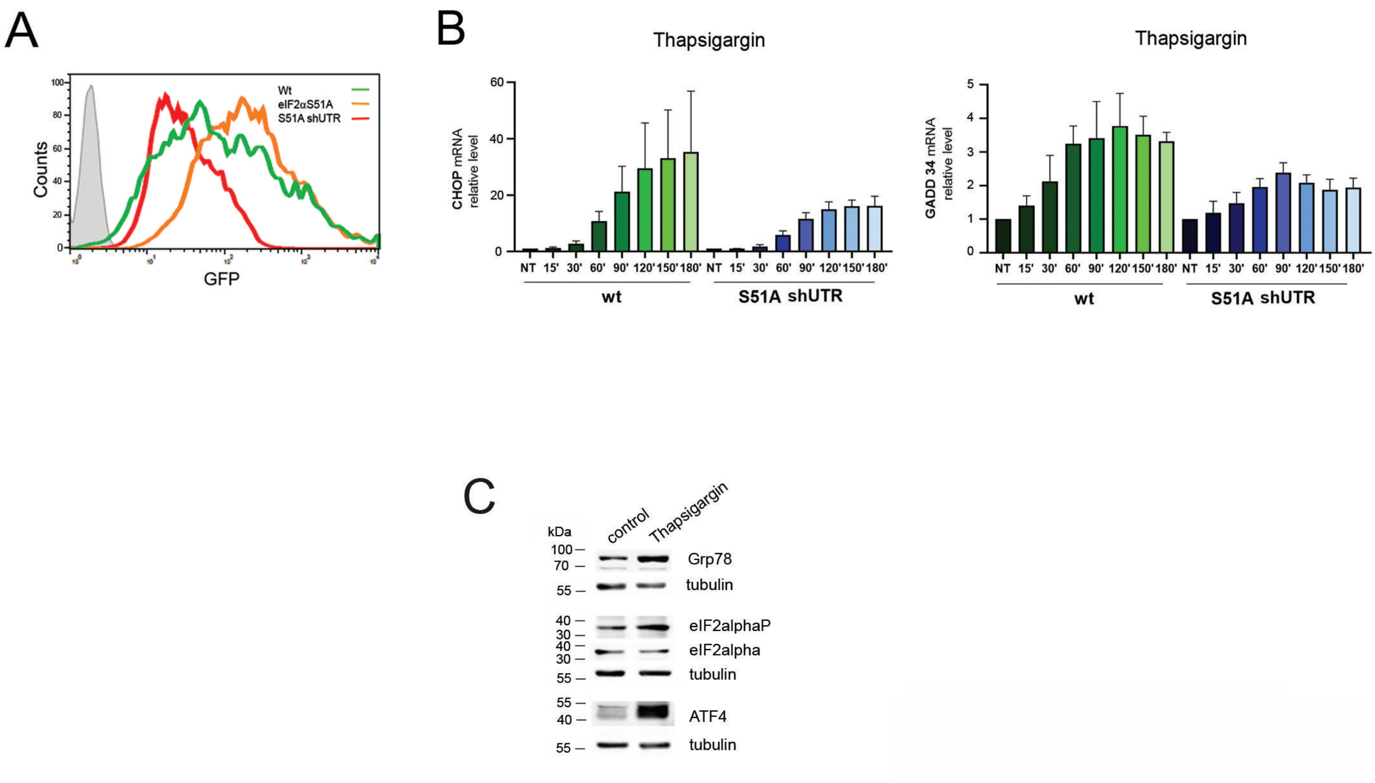

A. GFP expression in wt, S51A and S51A shUTR K562 cells detected by flow cytometry. B. Expression levels of CHOP and GADD34 mRNAs measured by RT-qPCR in wt or S51A shUTR K562 cells preconditioned with 100nM thapsigargin for 2 hours to induce ISR. The level of not treated cells was used as a reference =1. Statistical analysis: unpaired Student's t-test with Welch's correction (*p ≤ 0.05; **p ≤ 0.005; ***p ≤ 0.0005).
C. ISR induction by pretreatment with 100 nM thapsigargin. K562 cells were incubated with thapsigargin for 16 hours. Protein levels of Grp78, eIF2alphaP, eIF2alpha and ATF4, together with tubulin as loading control are shown.
The full-length membranes were properly cut based on the protein marker size according to target protein sites prior to hybridization with primary antibodies (see Materials and Methods). Cropped blots are presented; original non-cropped membranes are shown in the Supplementary Fig. S9.

Supplementary Figure 2.
Effects of imatinib and ISRIB on prosurvival signaling in CML cells

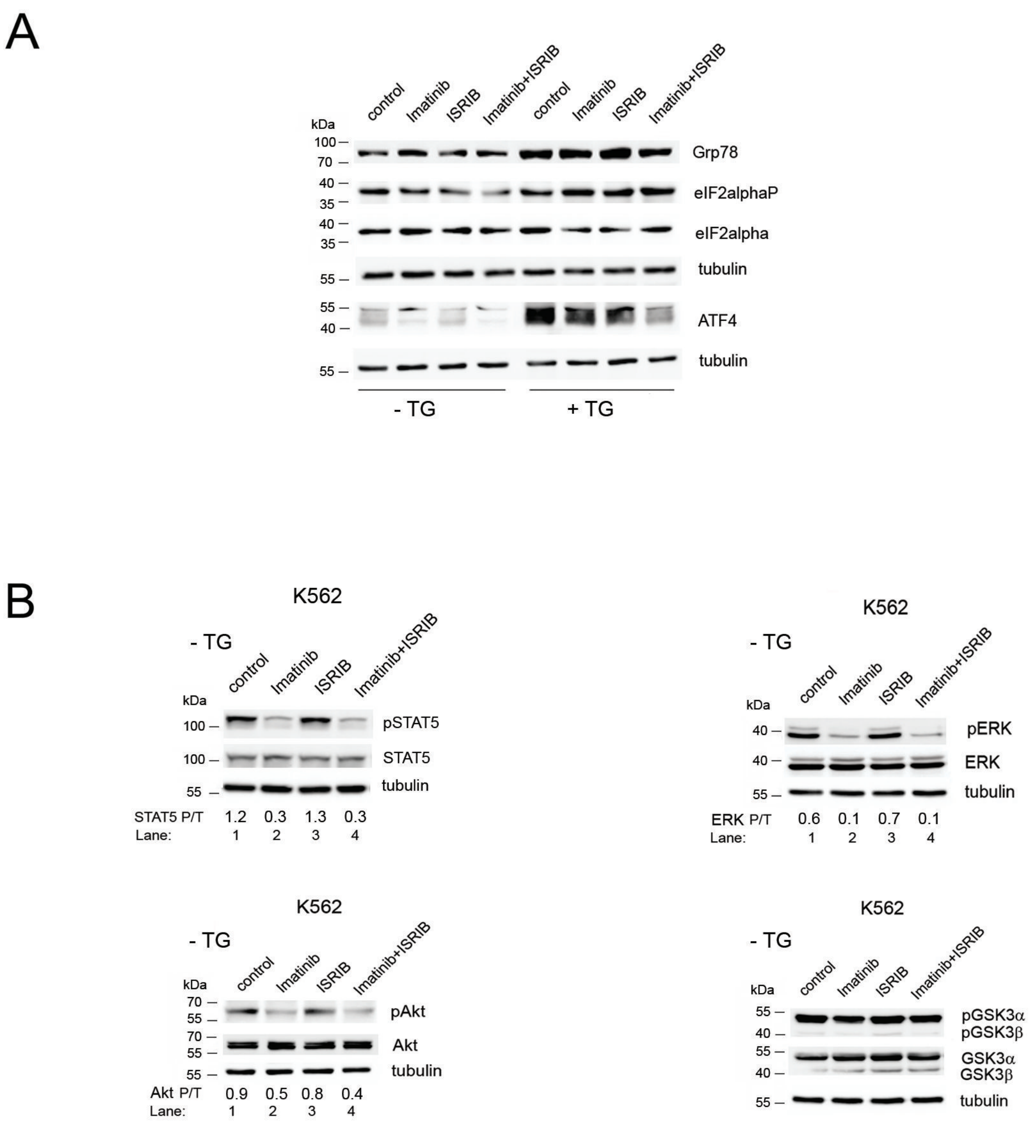

A. Effects of ISRIB and imatinib treatment on ISR signaling in CML cells without (-TG) or with (+TG) thapsigargin to activate ISR *in vitro.* If indicated (ISRIB alone and imatinib+ISRIB conditions), 250 nM ISRIB was added for 2 hours. Then, if indicated (+TG), 100 nM thapsigargin was added for 2 hours to mimic activation of ISR *in vitro*. This was followed by treatment with 1 μM imatinib (imatinib alone and imatinib+ISRIB conditions). After 16 hours cells were collected for analyses. The levels of Grp78, eIF2alpa and phosphorylated form of eIF2alpha (eIF2alphaP) and ATF4 were detected by western blot. Tubulin was used as a loading control.

B. Protein levels of STAT5 and phosphorylated STAT5 (pSTAT5), ERK and phosphorylated ERK (pERK), AKT and phosphorylated AKT (pAKT) and GSKα/β and phosphorylated GSKα/β (pGSKα/β) in wt K562 cells not pretreated with thapsigargin (-TG) and treated with imatinib and ISRIB as indicated, detected by western blot. Tubulin was used as a loading control. The ratio of phosphorylated to total forms (P/T) calculated based on the densitometry signal is given for each condition. A,B. The full-length membranes were properly cut based on the protein marker size according to target protein sites prior to hybridization with primary antibodies (see Materials and Methods). Cropped blots are presented; original non-cropped membranes are shown in the Supplementary Fig. S9.

Supplementary Figure 3.

Effect of combined treatment on BCR-ABL1 level, activity and cell viability.

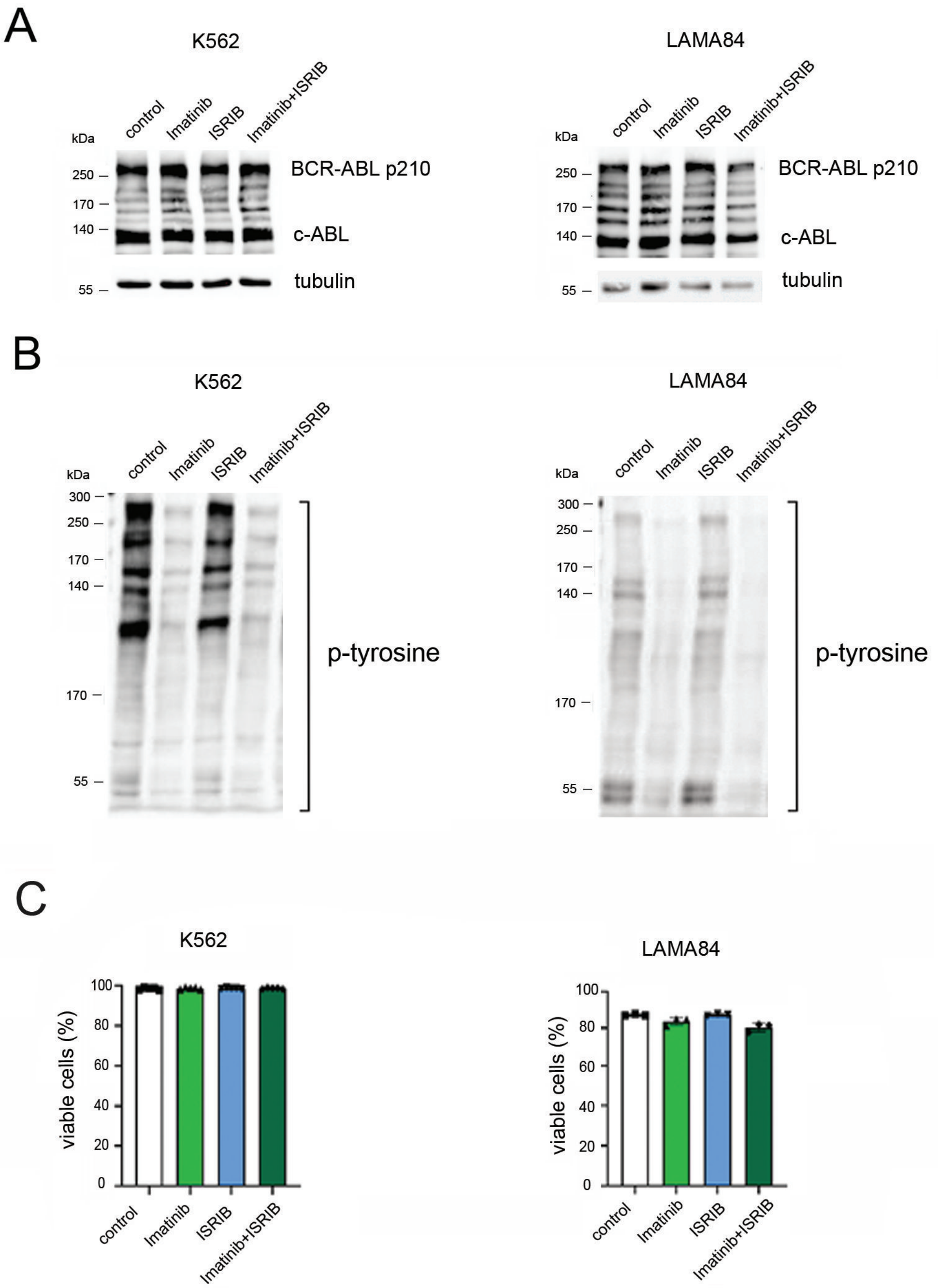

The levels of c-ABL and BCR-ABLp210 proteins (A) or phospho-tyrosines (B) detected in K566 and LAMA84 cells untreated (control) or treated as indicated. Tubulin was used as a loading control.
A, B. The full-length membranes were properly cut based on the protein marker size according to target protein sites prior to hybridization with primary antibodies (see Materials and Methods).
Cropped blots are presented; original non-cropped membranes are shown in the Supplementary Fig. S9
C - Percentage of viable cells detected by flow cytometry in control cells or treated as indicated.
For A, B, C cells were pretreated with 100 nM thapsigargin.

Supplementary Figure 4.
The Gating strategy for identification of engrafted human CD45+ (hCD45+) primary CML cells.

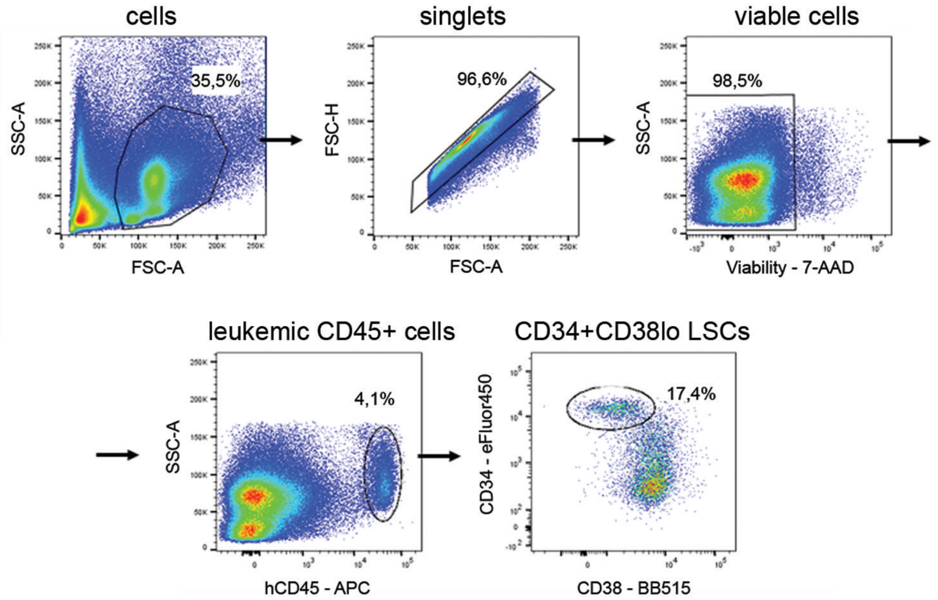

Representative dot plots showing cell gate (FSC-A/SSC-A), doublets discrimination – singlets gate (FSC-A/FSC-H), viability gate (7-AAD/SSC-A) and hCD45+ cells (hCD45-APC/SSC-A). Percentages of cells in each gate in the representative experiment are shown.

Supplementary Figure 5A.
Genes displaying significant difference in expression in pairwise treatment conditions.

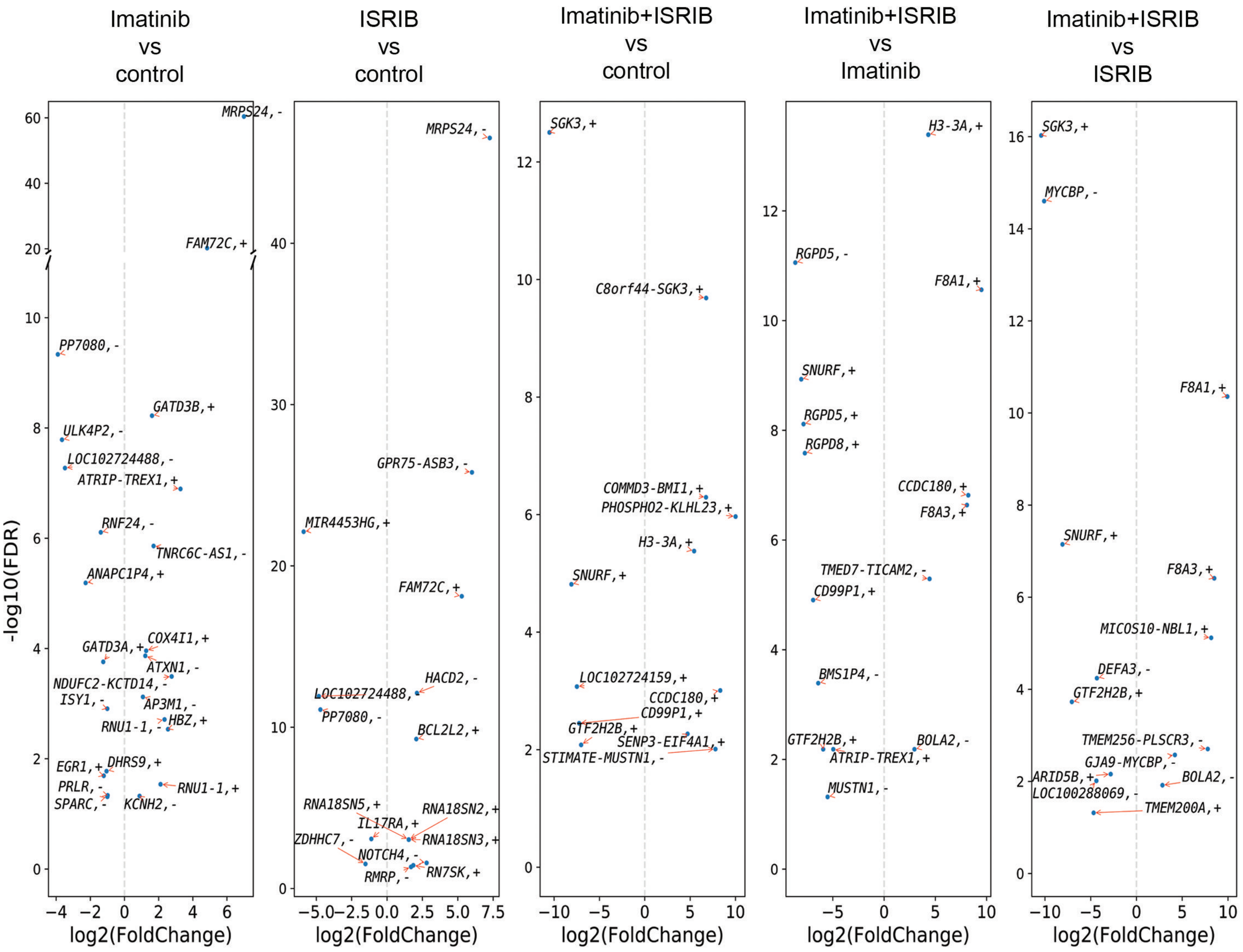

Fold changes in gene expression (log2FoldChange) vs corrected P-values (-log10FDR) are shown for indicated pairwise comparisons. The significance threshold of FDR 5% has been applied.

Supplementary Figure 5B.
Functional enrichment analysis of co-expressed gene clusters: significantly enriched REACTOME terms shown.

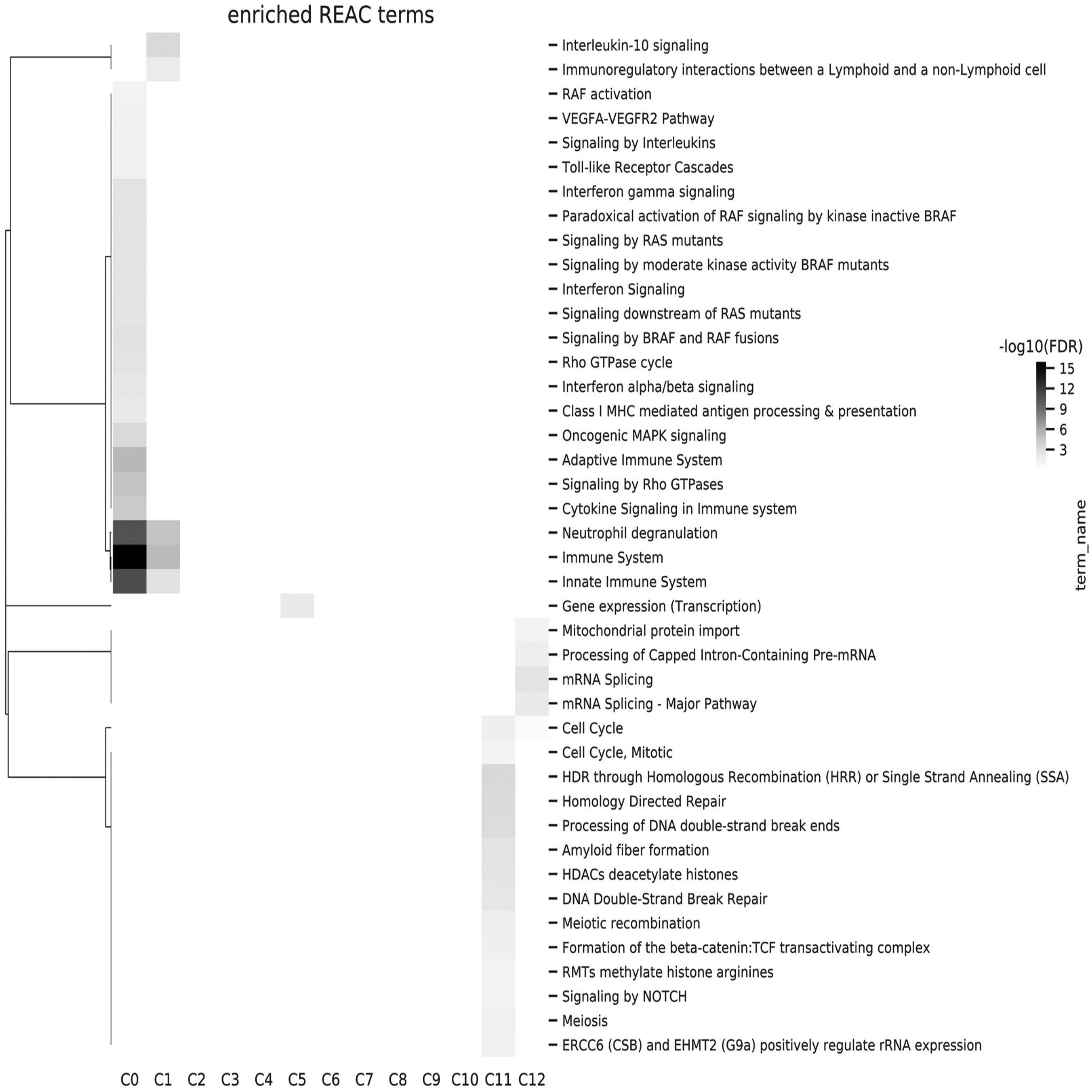

Significantly enriched REACTOME terms in all clusters (C0-C12) of co-expressed genes are presented. Transcriptome analysis has been done on hCD45+ cells isolated from PDX mouse model with CD34+ CML-BP imatinib resistant blasts.

Supplementary Figure 6. Gene Ontology Biological Processes (BP) analysis of co-expressed genes.

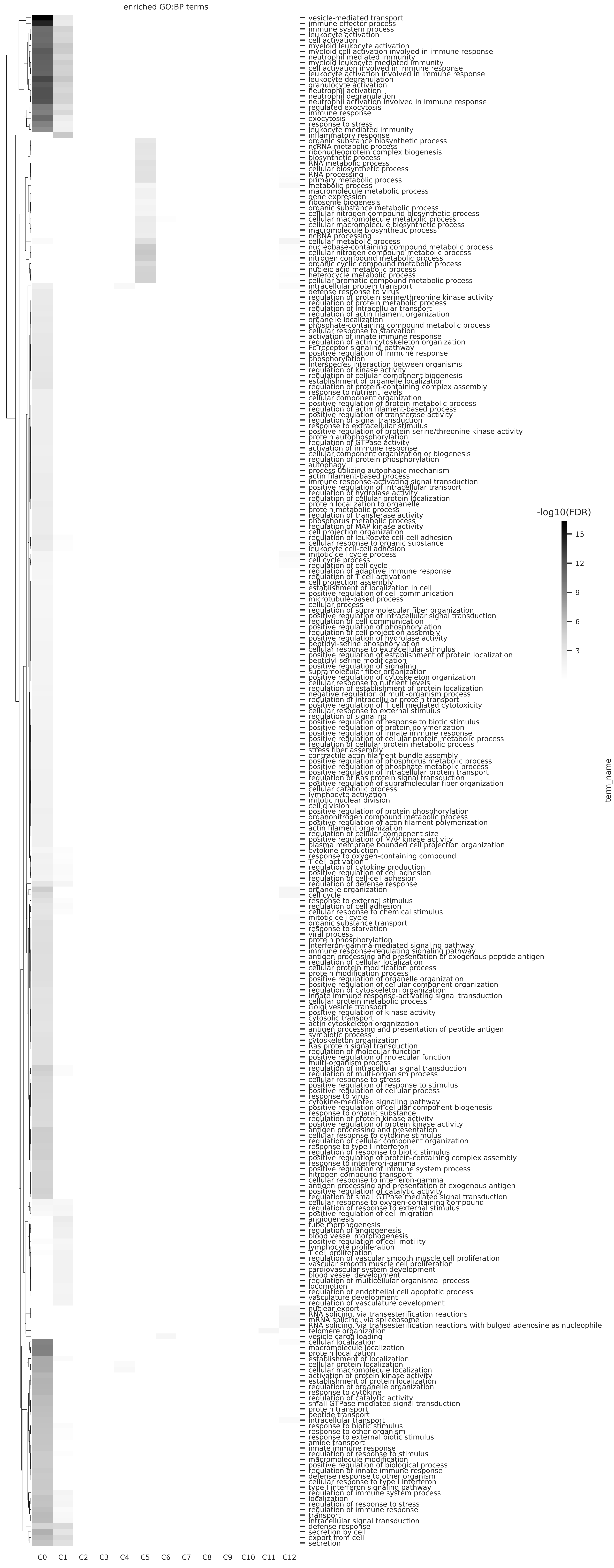

Significantly enriched GO:BP terms for all clusters (C0-C12) of co-expressed genes are presented. Transcriptome analysis has been done on hCD45+ cells isolated from PDX mouse model with CD34+ CML-BP imatinib resistant blasts.

Supplementary Figure 7. Expression changes of SGK-3 interaction partner genes.

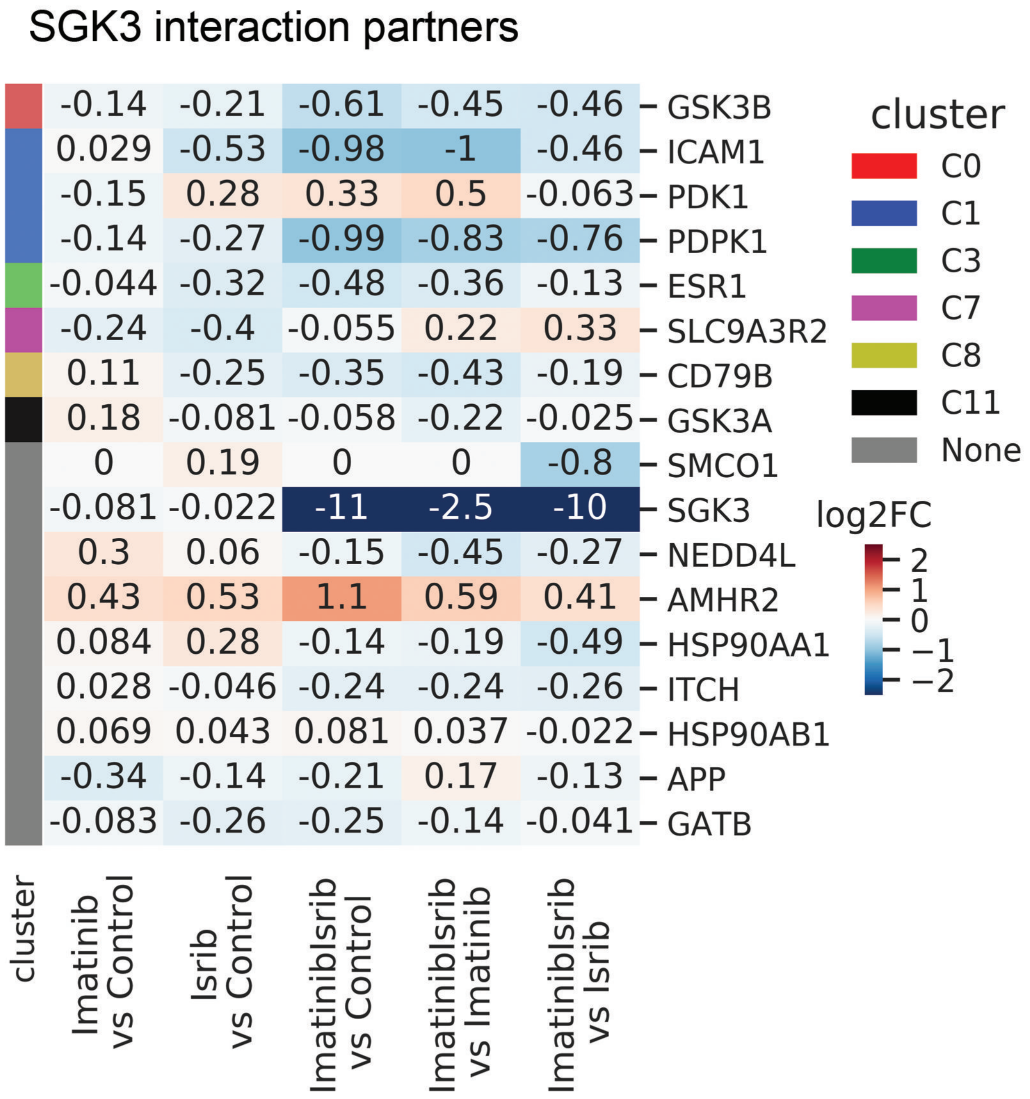

Heatmap showing changes in expression levels (log2FoldChange) of selected SGK3 interaction partner genes across indicated treatment comparisons. The membership of these genes to co-expression clusters are indicated in the figure legend ("None" if not a member of any co-expression cluster). Transcriptome analysis has been done on hCD45+ cells isolated from PDX mouse model with CD34+ CML-BP imatinib resistant blasts.

Supplementary Figure 8.
Expression of STAT5-target genes across treatment conditions.

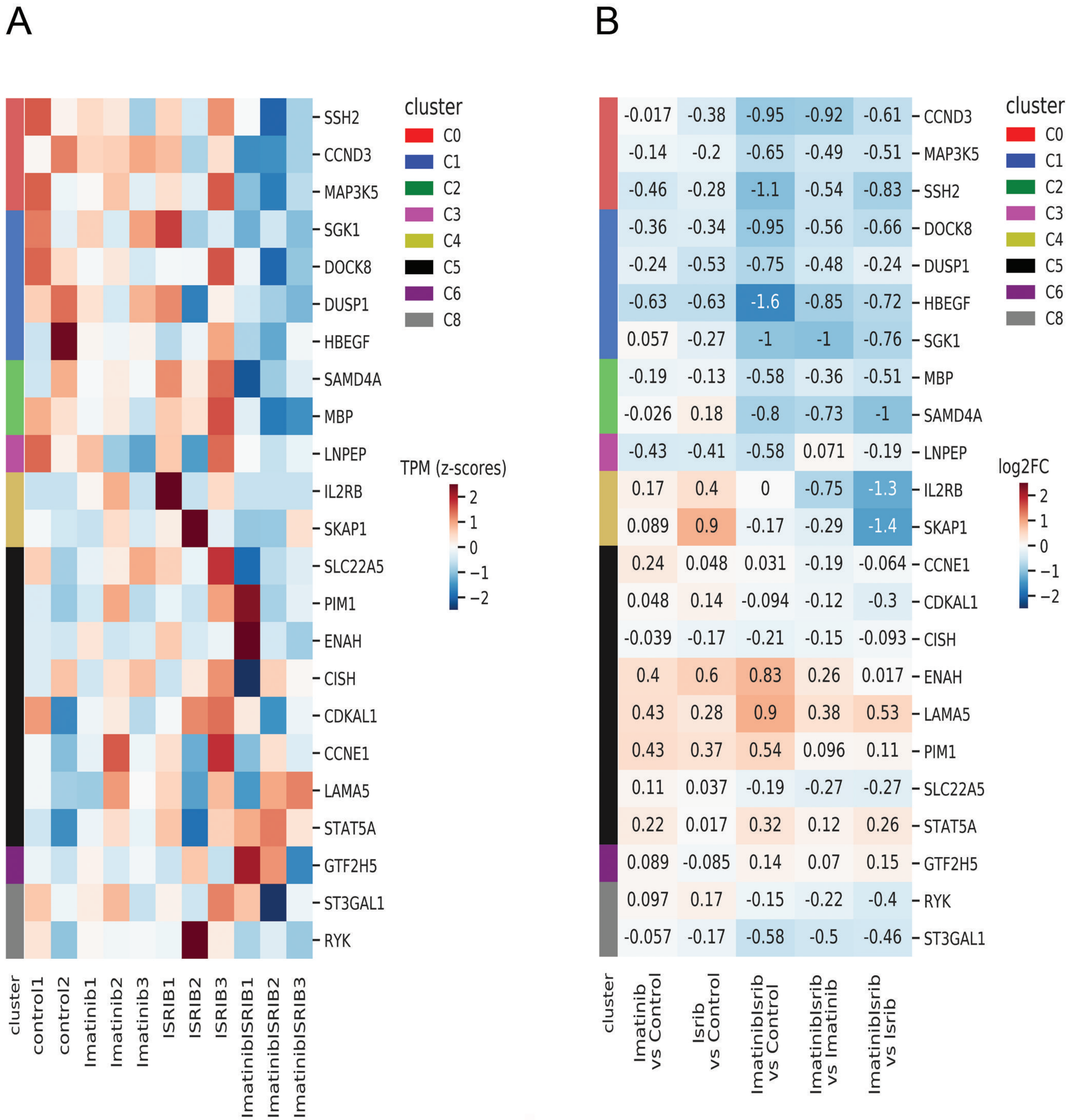

A. The heatmap showing expression level (transcripts per kilobase million or TPM, standardized with z-score) of each gene across all replicates of control (not treated) and treatments for STAT5-target genes.
B. The change in expression of target genes (log2FoldChange) for all treatment comparisons. The membership of these genes to co-expression clusters are indicated in the figure legend

Supplementary Figure 9.
Images of uncropped western blot membranes.

The full-length membranes were properly cut based on the protein marker size according to target protein sites into several parts prior to hybridization with primary antibodies, and every blot was then incubated with its primary antibody. Images of original non-cropped parts of membranes (after initial cut based on marker size prior the hybridization), together with different expositions are presented. If visualization of the membrane edges was not possible due to very strong signal (like for tubulin), they have been marked by frame.

Figure 1A

UNCROPPED:

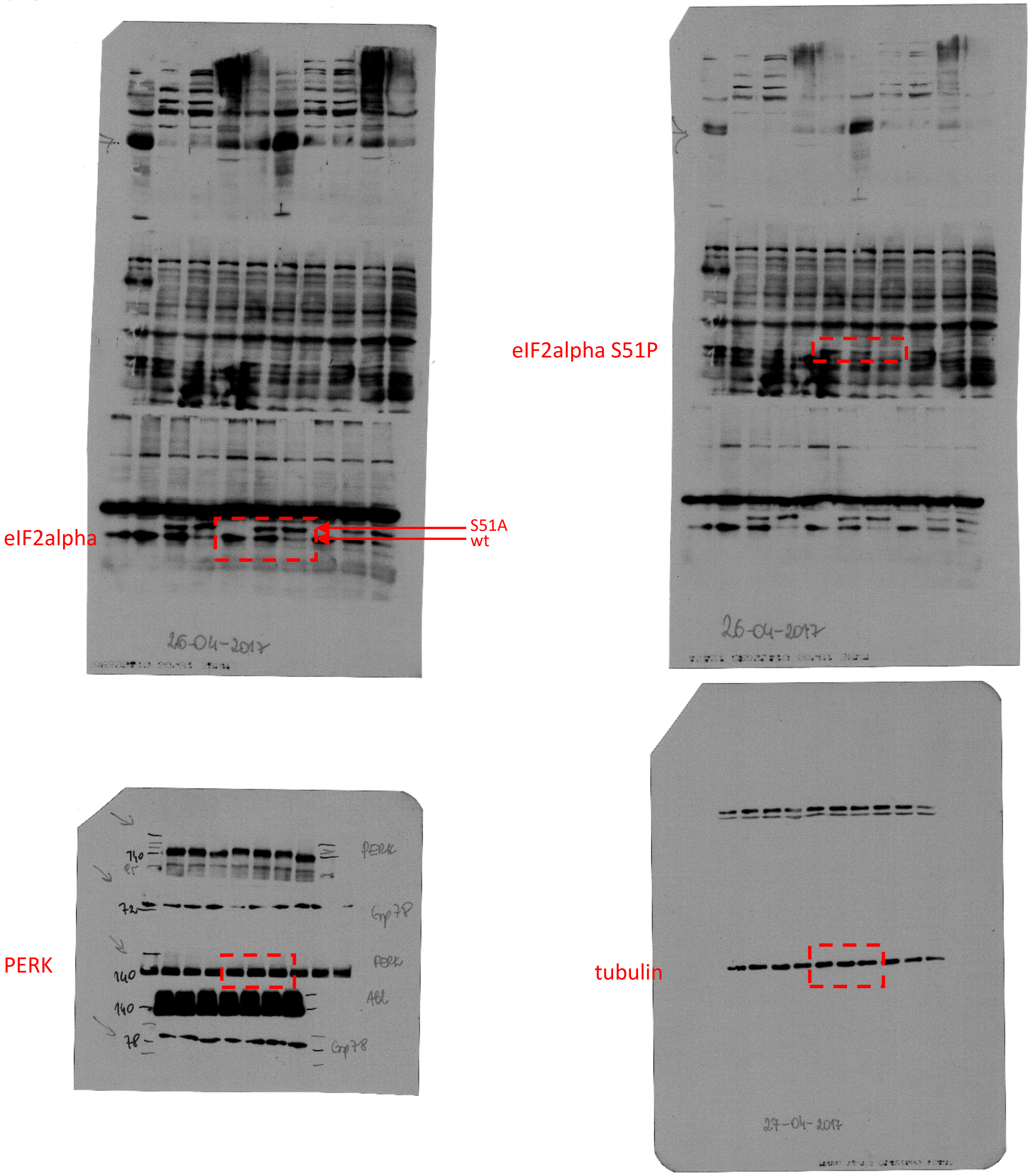

CROPPED:

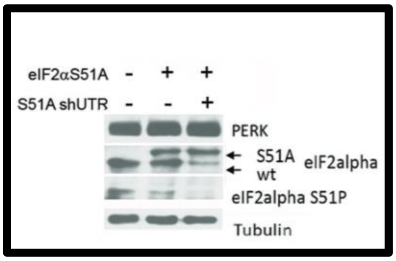

Figure 2A (K562 cells)

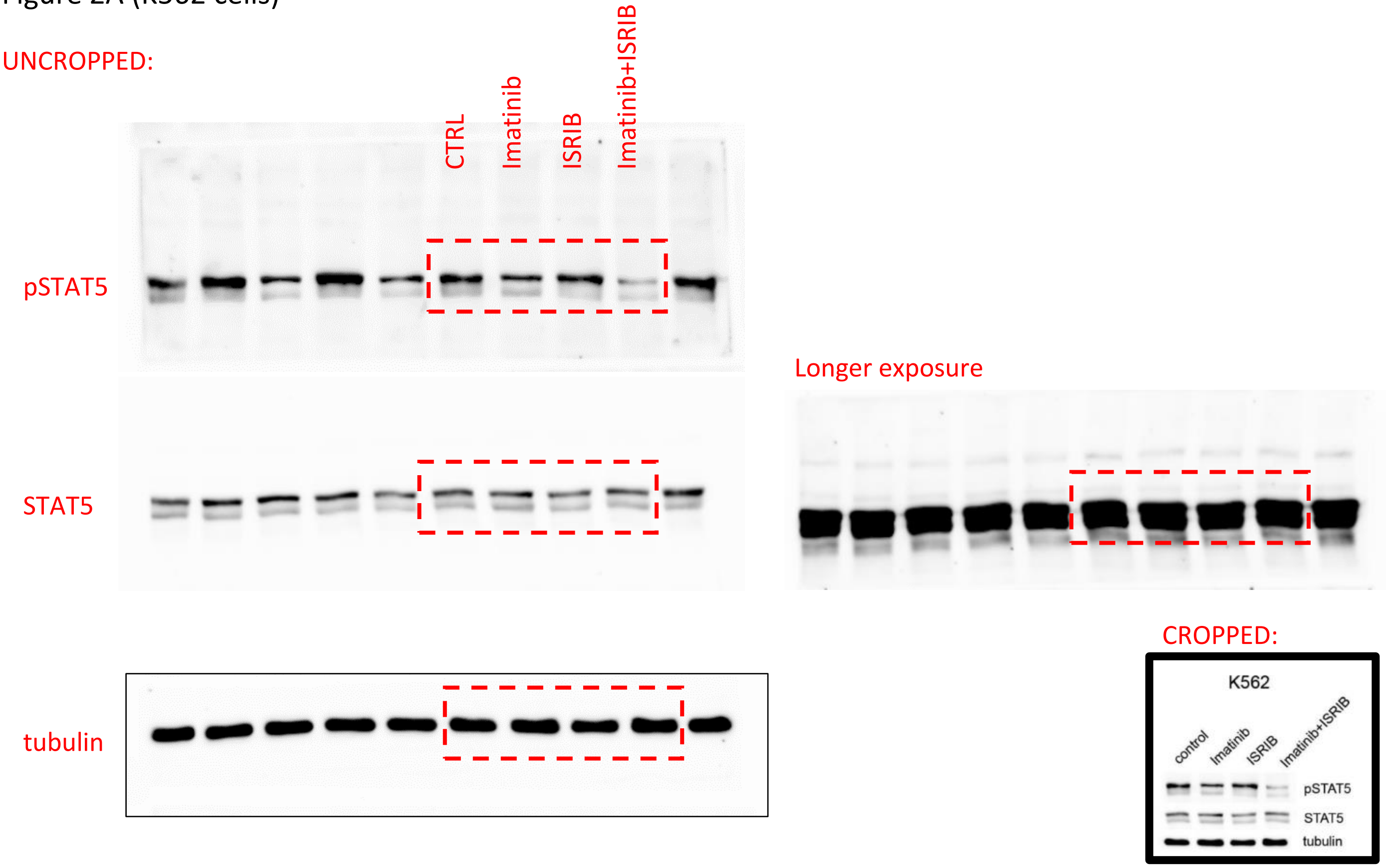

Figure 2A (LAMA84 cells)

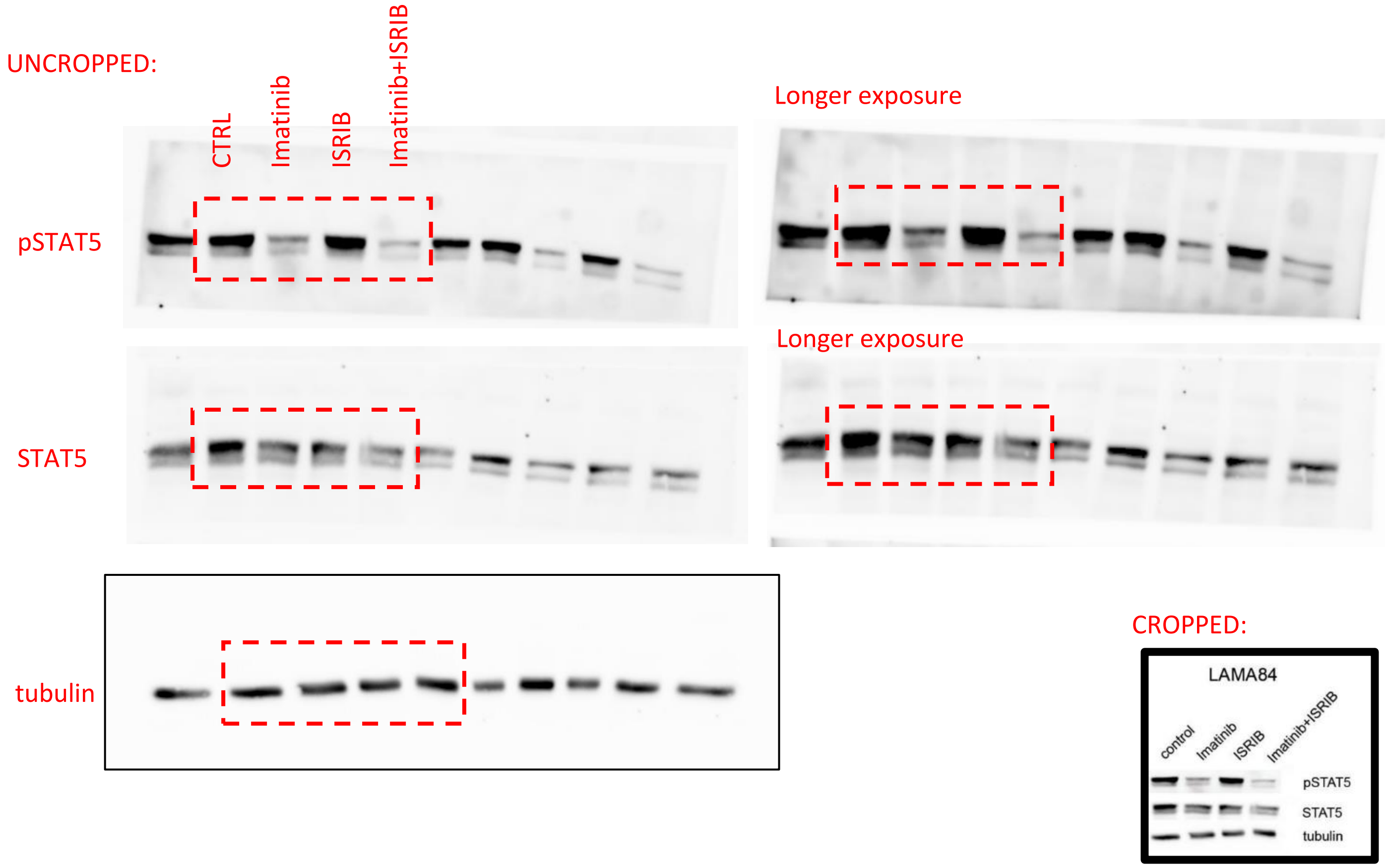

Figure 2C (K562 cells)

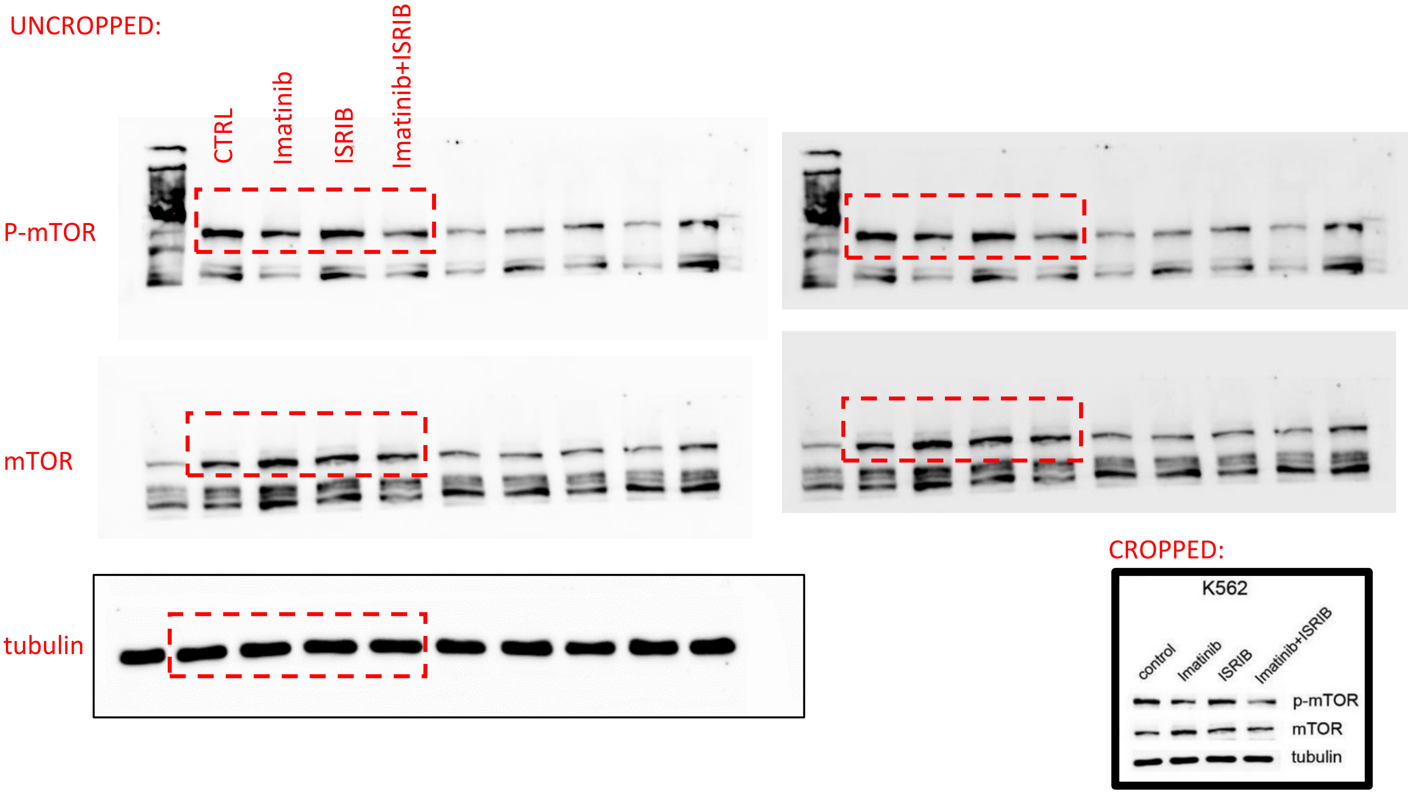

Figure 2C (LAMA84 cells)

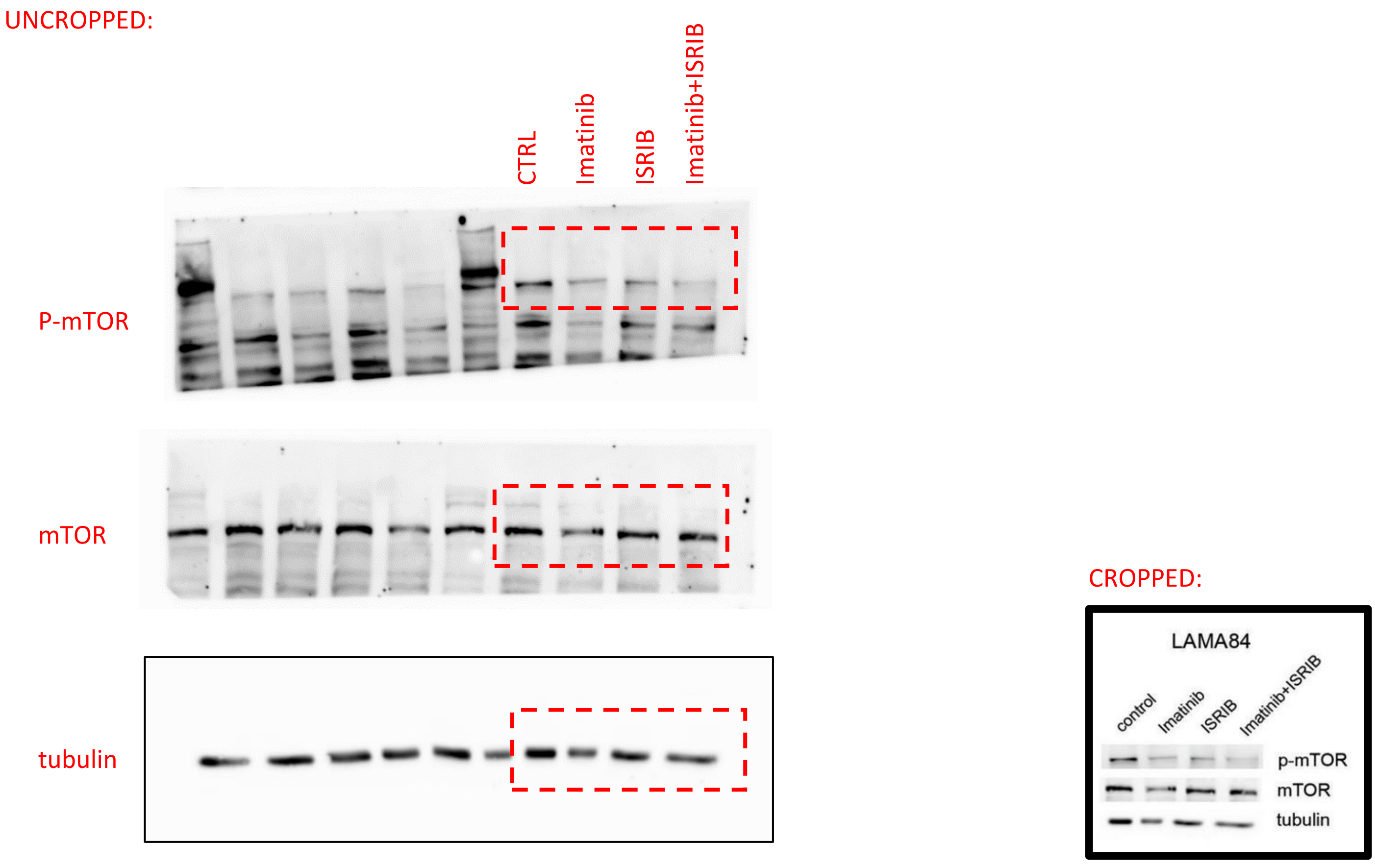

Figure 2E (K562 cells)

UNCROPPED:

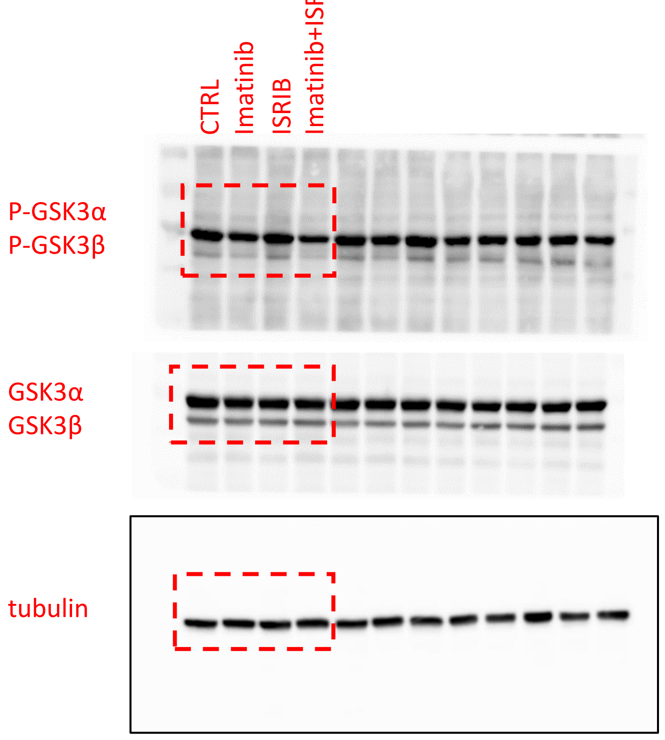

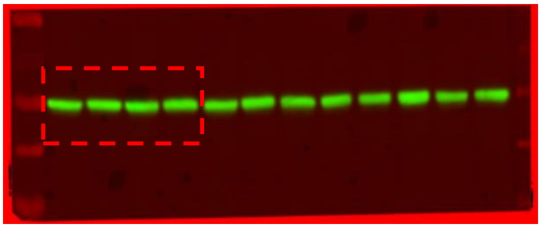

CROPPED:

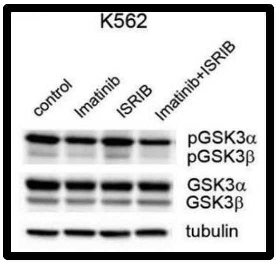

Figure 2E (LAMA84 cells)

UNCROPPED:

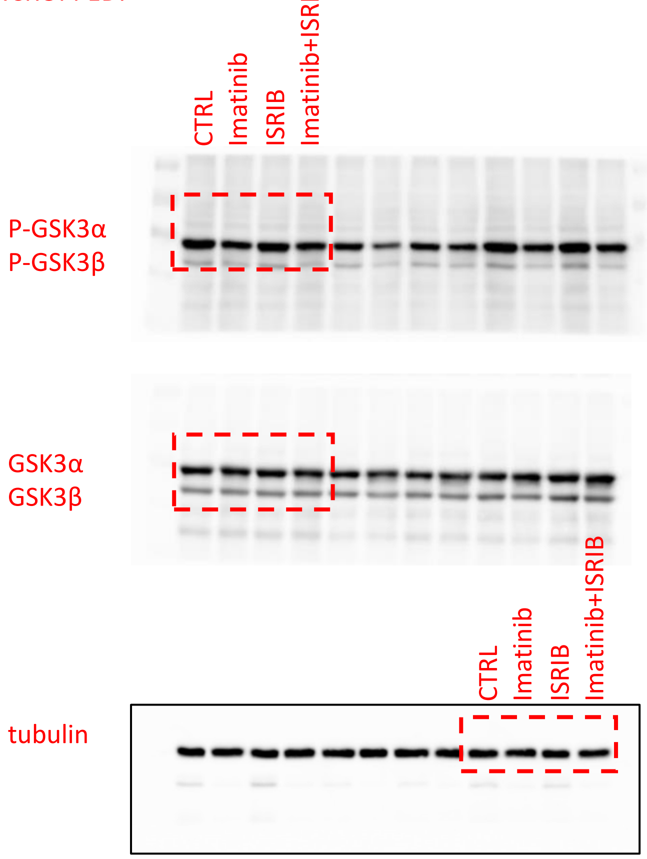

CROPPED:

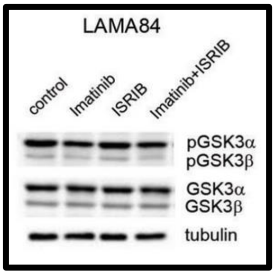

Figure 2F (K562 cells)

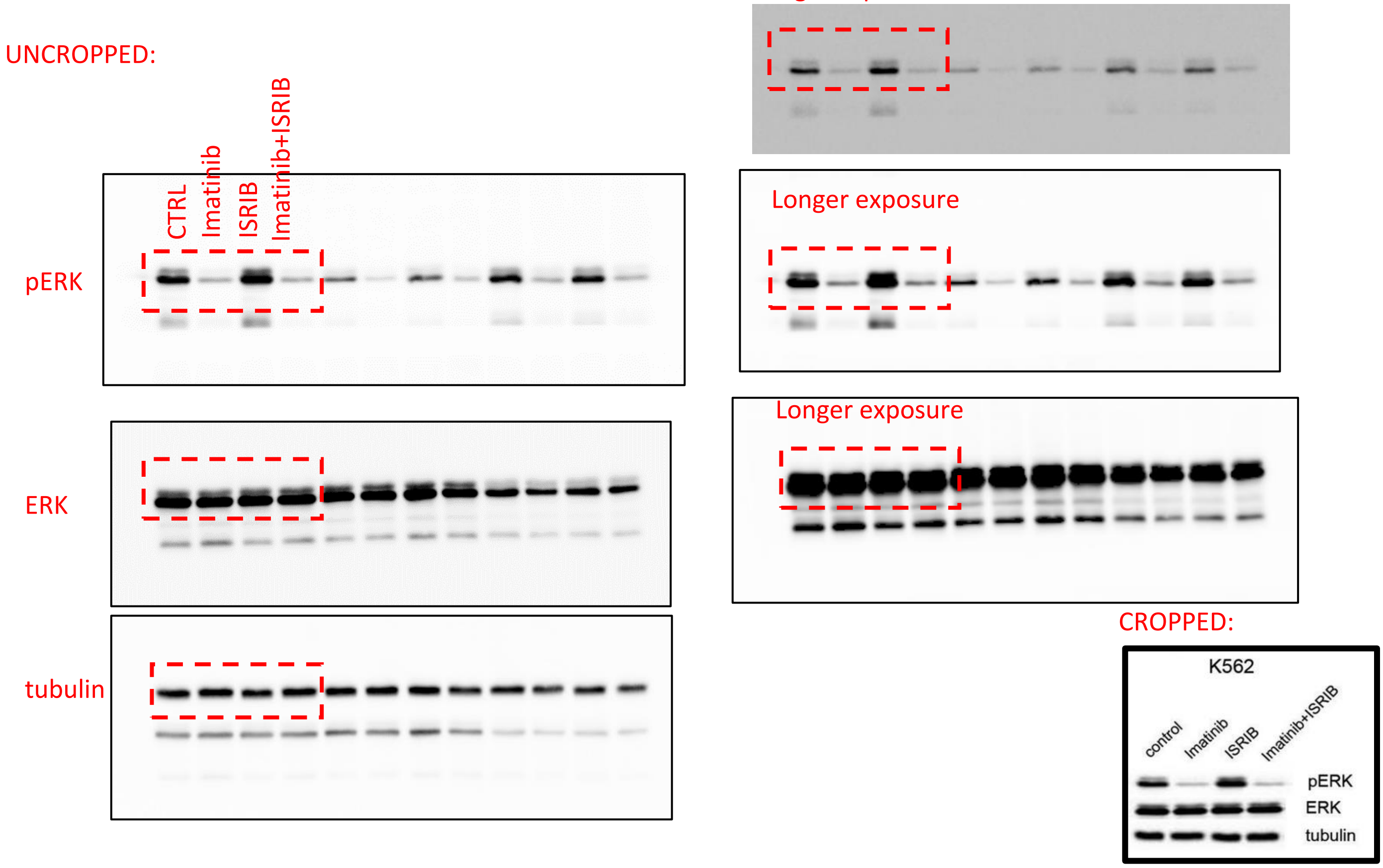

Figure 2F (LAMA84 cells)

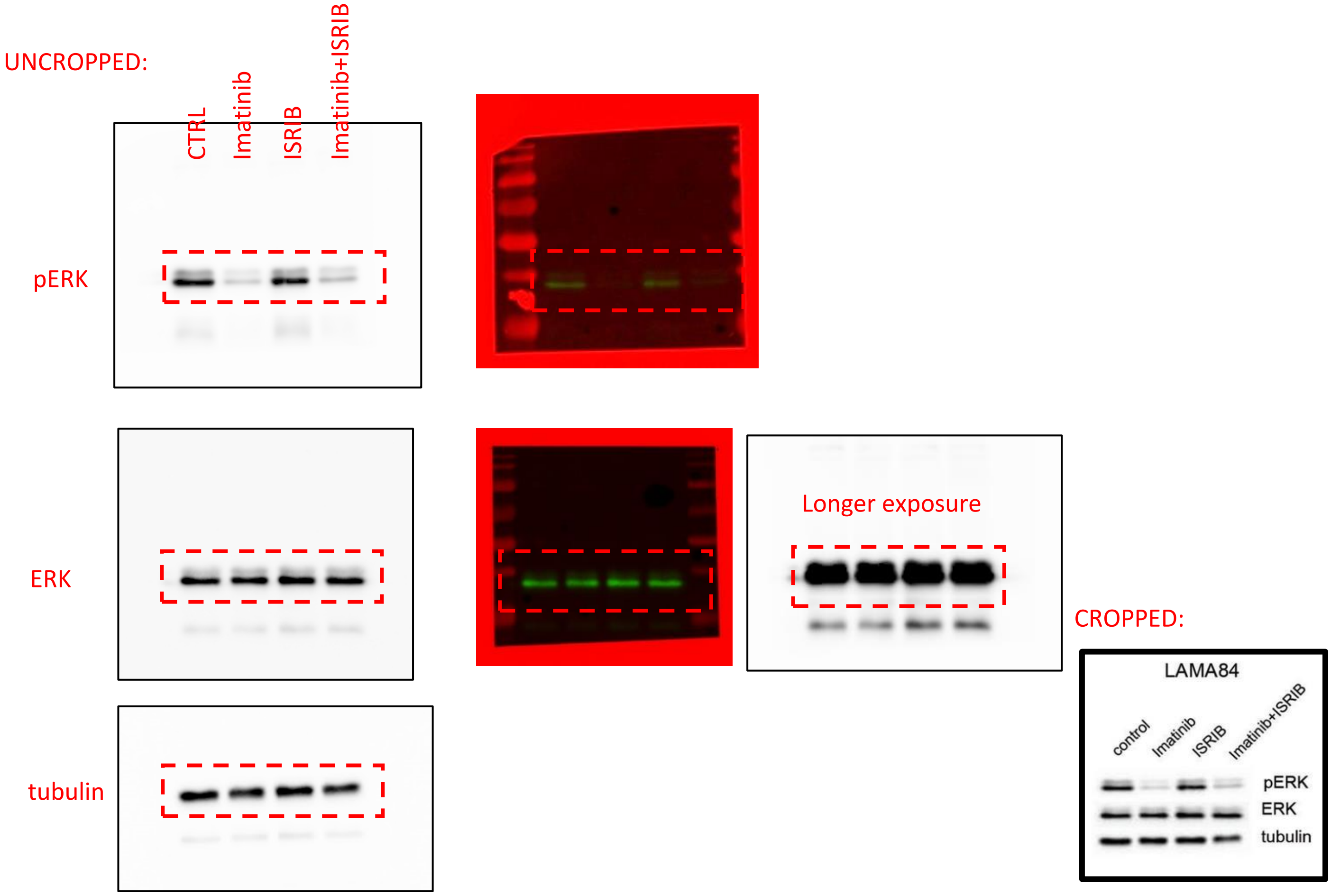

Figure 2G (K562 cells)

UNCROPPED:

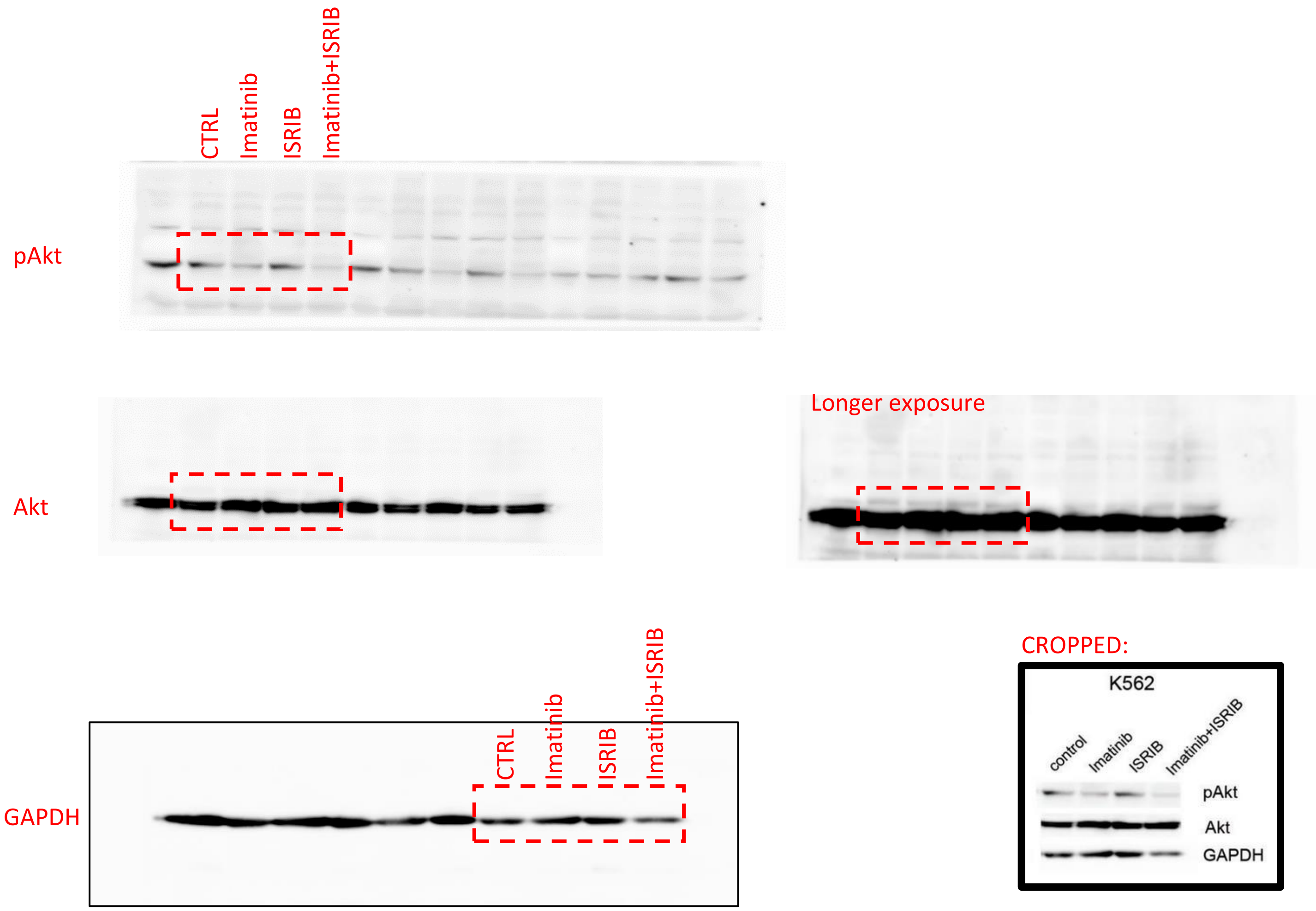

Figure S1C

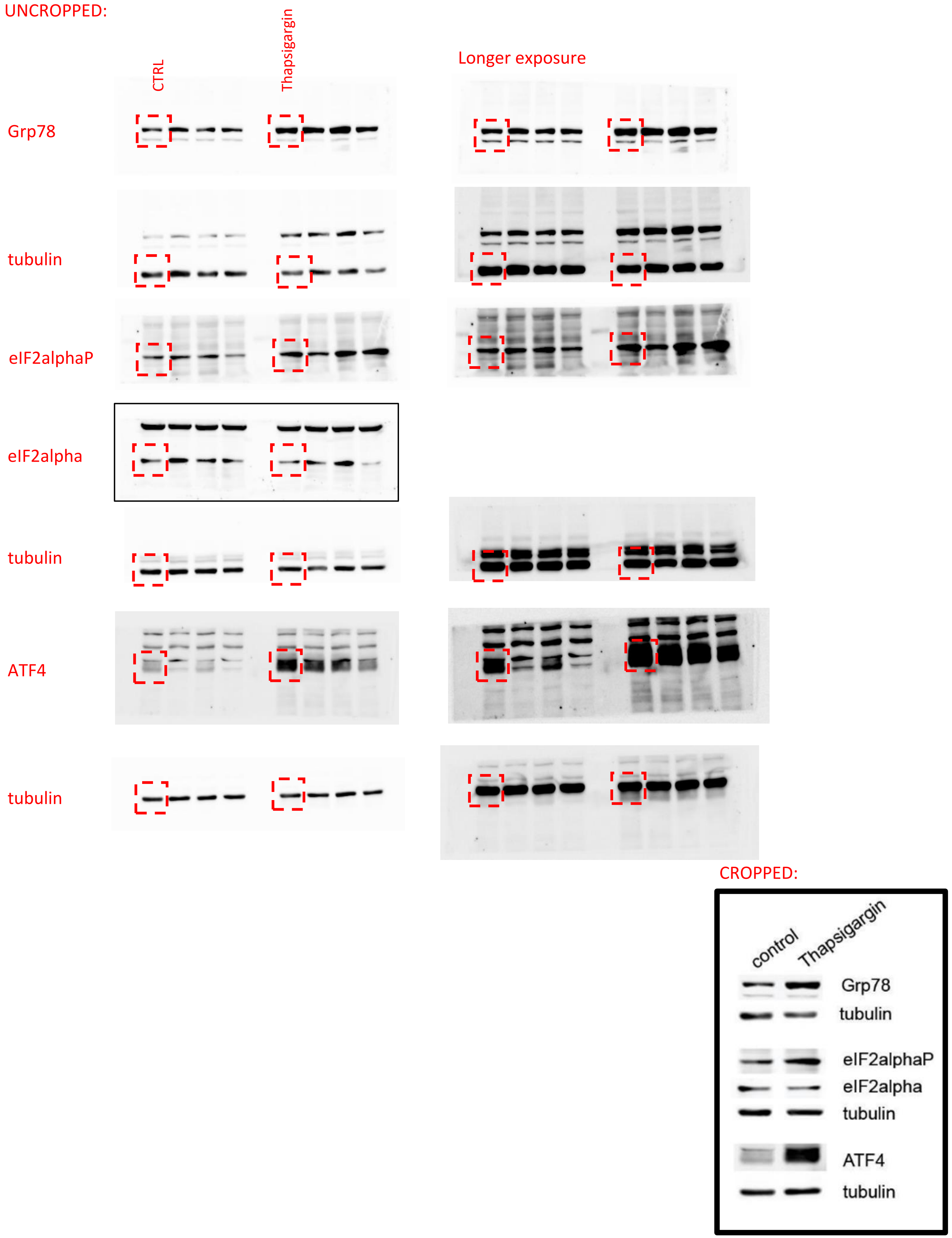
UNCROPPED:
CTRL
Thapsigargin
Longer exposure
Grp78
tubulin
eIF2alphaP
eIF2alpha
tubulin
ATF4
tubulin
CROPPED:
control
Thapsigargin
Grp78
tubulin
eIF2alphaP
eIF2alpha
tubulin
ATF4
tubulin

Figure S2A

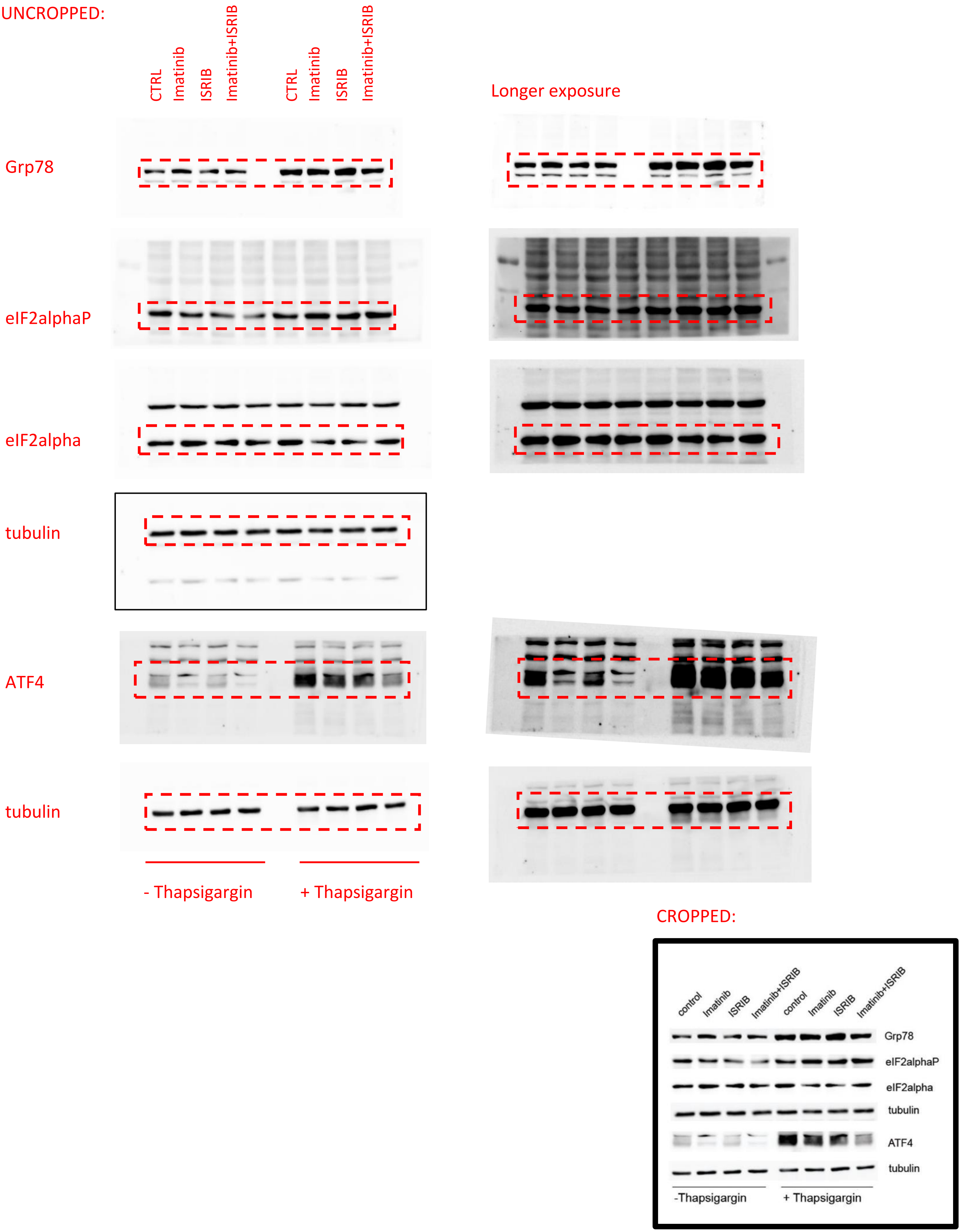
UNCROPPED:
CTRL
Imatinib
ISRIB
Imatinib+ISRIB
CTRL
Imatinib
ISRIB
Imatinib+ISRIB
Longer exposure
Grp78
eIF2alphaP
eIF2alpha
tubulin
ATF4
tubulin
- Thapsigargin
+ Thapsigargin
CROPPED:
control
Imatinib
ISRIB
Imatinib+ISRIB
control
Imatinib
ISRIB
Imatinib+ISRIB
Grp78
eIF2alphaP
eIF2alpha
tubulin
ATF4
tubulin
-Thapsigargin
+ Thapsigargin

Figure S2B

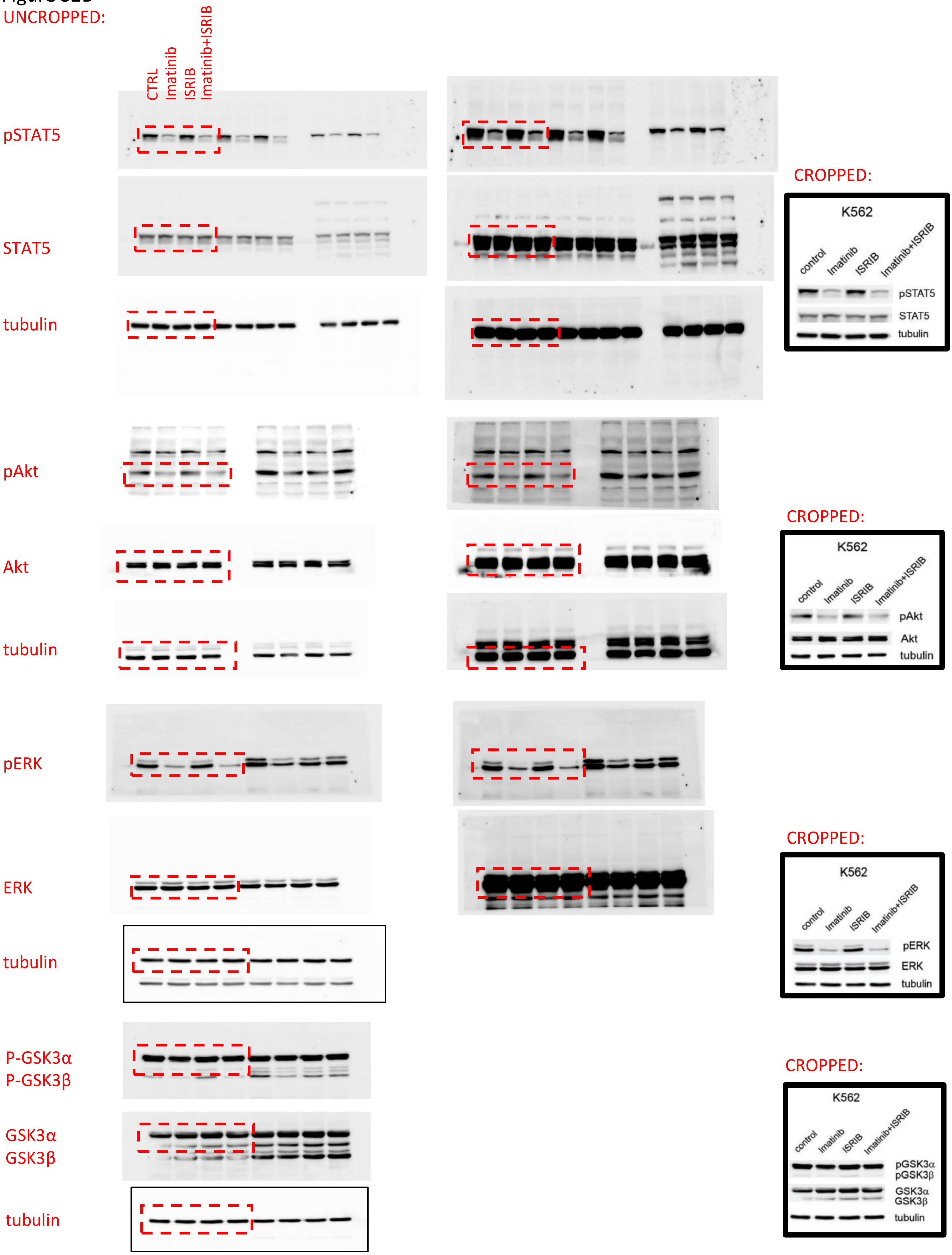
UNCROPPED:
CTRL
Imatinib
ISRIB
Imatinib+ISRIB
pSTAT5
STAT5
tubulin
pAkt
Akt
tubulin
pERK
ERK
tubulin
P-GSK3α
P-GSK3β
GSK3α
GSK3β
tubulin
CROPPED:
K562
control
Imatinib
ISRIB
Imatinib+ISRIB
pSTAT5
STAT5
tubulin
CROPPED:
K562
control
Imatinib
ISRIB
Imatinib+ISRIB
pAkt
Akt
tubulin
CROPPED:
K562
control
Imatinib
ISRIB
Imatinib+ISRIB
pERK
ERK
tubulin
CROPPED:
K562
control
Imatinib
ISRIB
Imatinib+ISRIB
pGSK3α
pGSK3β
GSK3α
GSK3β
tubulin

Figure S3A (K562 cells)

UNCROPPED:

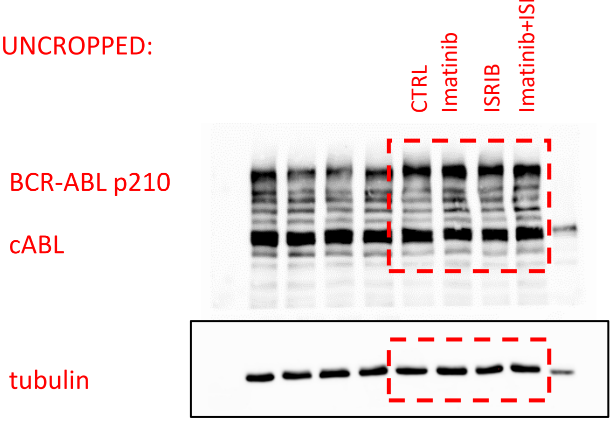

CROPPED:

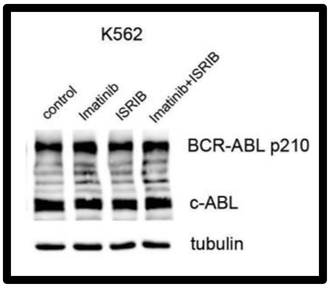

Figure S3A (LAMA84 cells)

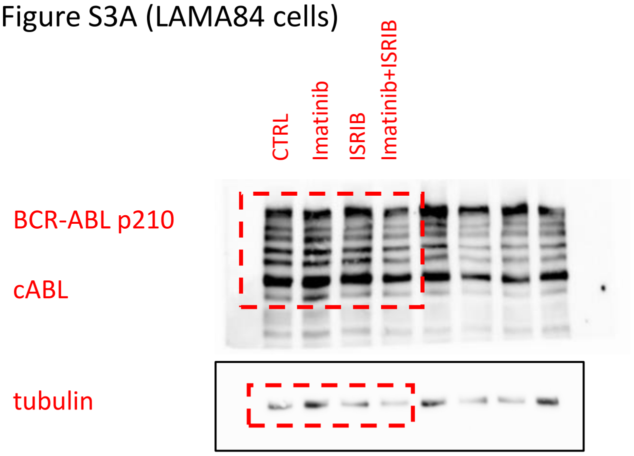

CROPPED:

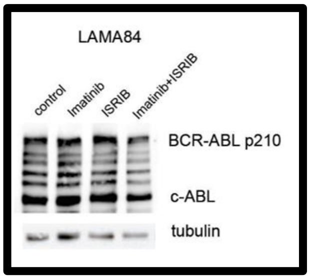

Figure S3B (K562 cells)

UNCROPPED:

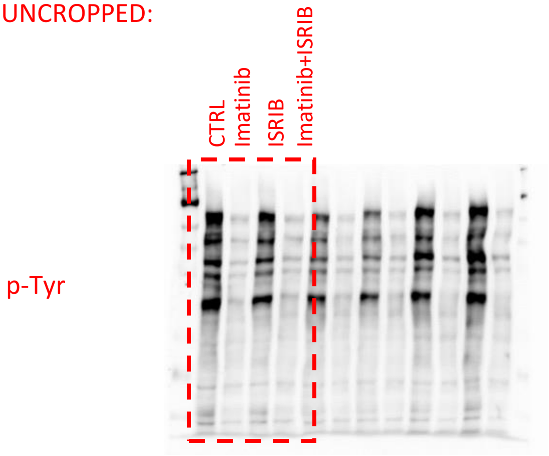

CROPPED:

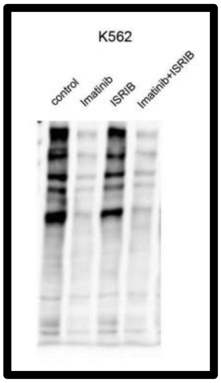

Figure S3B (LAMA84 cells)

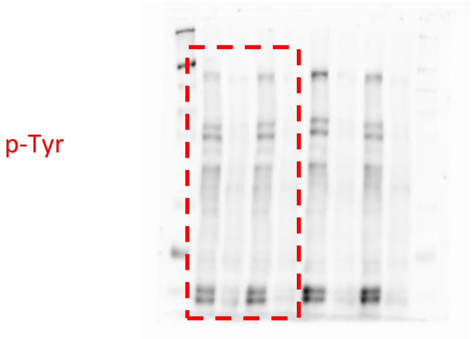

CROPPED:

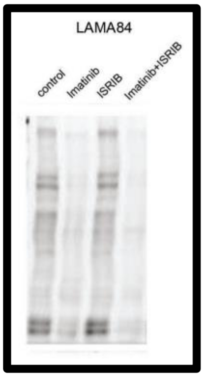

Supplement: Supplementary file 1 — Additional file 1. [file 12885_2022_10289_MOESM1_ESM.pdf]
